# FGF receptor kinase inhibitors exhibit broad antiviral activity by targeting Src family kinases

**DOI:** 10.1007/s00018-024-05502-x

**Published:** 2024-12-02

**Authors:** Debora Stefanova, Dominik Olszewski, Mirco Glitscher, Michael Bauer, Luca Ferrarese, Daria Wüst, Eberhard Hildt, Urs F. Greber, Sabine Werner

**Affiliations:** 1 Institute of Molecular Health Sciences, Department of Biology, ETH Zürich, Otto-Stern-Weg 7, Zürich, 8093 Switzerland; 2 Department of Molecular Life Sciences, University of Zürich, Winterthurerstrasse 190, Zurich, 8057 Switzerland; 3 Paul-Ehrlich-Institute, Department of Virology, D-63225 Langen, Germany

**Keywords:** FGF, FGFR, Interferon response genes, Viral infection, Src kinase, Lyn kinase

## Abstract

The development of antiviral strategies is a key task of biomedical research, but broad-spectrum virus inhibitors are scarce. Here we show that fibroblast growth factor receptor (FGFR) tyrosine kinase inhibitors reduce infection of several cell types with DNA and RNA viruses by blocking early stages of infection, but not viral cell association. Unexpectedly, their antiviral activity was largely independent of FGFR kinase inhibition. RNA profiling showed upregulation of interferon response genes by FGFR inhibitors, but their expression did not correlate with the antiviral activity in infected cells. Using bioinformatics analysis of kinome data, targeted kinase assays, siRNA-mediated knock-down and pharmacological inhibition experiments, we show that blockade of Src family kinases, in particular Lyn, is mainly responsible for the antiviral activity of FGFR inhibitors. These results identify FGFR inhibitors as broad-spectrum antiviral agents and suggest the poorly studied Lyn kinase as a promising target for the treatment of viral infections.

**Supplementary Information:**

The online version contains supplementary material available at 10.1007/s00018-024-05502-x.

## Introduction

Inhibition of FGFR signaling is a promising strategy for the treatment of various malignancies and of certain genetic diseases, which are associated with increased FGFR kinase activity, such as achondroplasia or Kallmann syndrome [1]. FGFR inhibitors target one or more of the four FGFR tyrosine kinases (FGFR1-4), which are activated by the large family of FGFs [2–4]. However, the use of FGFR inhibitors is a double-edged sword, since they can have a beneficial therapeutic effect, but negatively impact tissue homeostasis and repair because of the widespread biological activities of FGFs [5, 6].

We recently showed that signaling through FGFRs modulates immune responses in keratinocytes. FGF treatment of mouse or human keratinocytes suppressed interferon-stimulated gene (ISG) expression under homeostatic conditions and in response to interferon exposure in a cell-autonomous manner. Vice versa, mice with a keratinocyte-specific knockout of *Fgfr1* and *Fgfr2* showed increased expression of ISGs in the epidermis [7]. This is functionally relevant, because activation of FGFR2 signaling in keratinocytes by its canonical ligands FGF7 and FGF10 promoted infection with Herpes Simplex Virus (HSV)-1 or Zika virus, which correlated with reduced expression of ISGs [7]. Consistently, a role of FGFR signaling in the control of viral infections has been shown for several other cell types and viruses [8]. Endogenous FGFs may be important for this effect, because secretion of different FGFs was promoted during Zika virus infection and in HSV-1 infected mouse brain neurons [9, 10]. Furthermore, subcutaneous injection of HSV-1 into mouse back skin induced the expression of *Fgf7* [7].

In most studies, stimulation of FGFR signaling suppressed immune responses and/or promoted susceptibility to infection with viruses, such as Zika and Influenza Virus, or bacteria, such as *Staphylococcus aureus* [9, 11–13]. However, some studies identified a beneficial effect of FGF treatment. For example, treatment with an engineered FGF1 reduced the severity of keratitis and blepharitis in HSV-1 infected mouse cornea without affecting viral replication [14], possibly because of the repair-promoting effect of FGF1. Furthermore, a few FGFs, which mainly activate FGFR3, inhibited infection of different cancer cell lines with Vesicular Stomatitis Virus or Coxsackie Virus B3 through as yet unidentified mechanisms that are independent of ISG regulation [15]. Thus, the role of FGFs in viral infection seems to depend on the infectious agent, the cell type, the type of involved FGFR and the timing of FGFR modulation.

Because most studies showed promotion of viral infections by FGFs, it is of major interest to determine if FGFR kinase inhibitors have antiviral activity. Indeed, we previously showed that treatment of immortalized human HaCaT keratinocytes with AZD4547 and BGJ398, which inhibit the kinase activity of FGFR1, FGFR2 and FGFR3 [16, 17], reduces the infection with HSV-1 [7]. Another FGFR kinase inhibitor suppressed infection of Huh-7.5 liver cancer cells with Hepatitis C Virus [18]. However, it is unclear if FGFR inhibitors also affect other viruses, if they are efficient in different cell types, including primary cells, and how they affect different stages of the infection cycle. In addition, it remains to be determined if their effect is entirely dependent on the inhibition of FGFR signaling, in particular at the relatively high doses of these compounds that are frequently used in vivo, e.g. for cancer treatment [19], or which were used for inhibition of viral infection in vitro [7, 18]. Finally, it is unclear if their antiviral activity results from upregulation of ISG expression.

Here we addressed these important questions with a focus on HSV-1, a double-stranded, enveloped DNA virus that belongs to the alpha subfamily of Herpes viruses [20]. It is a major cause of epithelial infections, affecting predominantly the oral mucosa. 45–90% of the population have been infected with this virus, with the highest infection frequency in the developing world [21]. In neonates or immunocompromised individuals, HSV-1 can cause serious life-threatening complications, such as herpes encephalitis [22]. Keratinocytes are an entry site for HSV-1 and the predominant cell type in which the virus replicates [23, 24]. Therefore, they are often utilized to study the biology of HSV infection.

We show here that several small molecule FGFR inhibitors promote resistance of keratinocytes to HSV-1 and also inhibit infection of different cell types with other viruses. Surprisingly, however, their broad antiviral activity was predominantly dependent on inhibition of Src family kinases, in particular Lyn kinase. These results offer interesting therapeutic opportunities and provide insight into the action of tyrosine kinase inhibitors in viral infection. They also reveal an important role of Lyn in the activity of some FGFR kinase inhibitors, which should be considered when these compounds are used for the treatment of cancer and other diseases.

## Materials and methods

### Cell lines and primary keratinocytes

The human immortalized, but non-transformed HaCaT keratinocyte cell line [25] was kindly provided by Dr. Petra Boukamp, Leibniz Institute for Environmental Research, Düsseldorf, Germany.

CaCo2 cells were purchased from Sigma-Aldrich, St. Louis, MO. HeLa cells were obtained from ATCC, Manassas, VA. Huh7 cells were kindly provided by Dr. Volker Thiel, University of Berne, Switzerland. All cell lines were cultured in DMEM/10% fetal bovine serum (FBS) for propagation, and in DMEM/5% FBS for viral infection studies. Absence of mycoplasma was confirmed by PCR using the PCR Mycoplasma Test Kit I/C (PromoKine, Heidelberg, Germany) on a monthly basis.

Human primary keratinocytes (HPK) were kindly provided by Dr. Hans-Dietmar Beer, University Hospital Zurich.

### Establishment and culture of mouse intestinal organoids

Mouse intestinal organoids were kindly provided by Drs. Annika Hausmann and Wolf-Dietrich Hardt (ETH Zurich). They were isolated and cultured as previously described [26]. Briefly, the organoids were embedded in Matrigel (Chemie Brunschwig, Basel, Switzerland) domes, covered with Complete Intesticult medium (Stemcell Technologies, Vancouver, Canada) and cultured at 37 °C/5% CO_2_. The medium was partially replaced every 3–4 days, and the organoids were sub-cultured upon mechanical shearing in Gentle Dissociation Reagent.

### Virus strains

HSV-1 was produced as described [7]. HSV-1 recombinant strain C12 expressing green fluorescent protein (GFP) under control of the major cytomegalovirus (CMV) promoter [27] was kindly provided by Dr. Stacey Efstathiou (University of Cambridge, UK). Encephalomyocarditis virus (EMCV) was kindly provided by Drs. Roman Spörri and Annette Oxenius (ETH Zurich, Switzerland). HCoV229E/GFP Gemini was kindly provided by Prof. Volker Thiel (University of Berne, Switzerland). In this virus, the open reading frame 4 had been replaced by the GFP cDNA [28]. Influenza Wisconsin (IAV WSN) strain was kindly provided by Dr. Silke Stertz (University of Zurich, Switzerland).

### Lactate dehydrogenase (LDH) activity quantification

LDH activity in the supernatant of treated cells was measured using the CyQUANT™ LDH Cytotoxicity Assay (C20300, Invitrogen, Waltham, MA) according to the manufacturer`s protocol. As per the protocol, addition of 10 µL water to the cells was used as negative control and addition of 10 µL lysis buffer provided with the kit was used as a positive control. The values for those were set to 0% and 100%, respectively, and used to calculate cytotoxicity levels in the test samples according to the manufacturer`s instructions.

### Tyrosine kinase inhibitors

The following inhibitors were used (all from Selleckchem, Houston, TX): FGFR1/2/3 inhibitors AZD4547 (S2801), BGJ398 (NVP-BGJ398; S2183), Erdafitinib (JNJ-42756493, S8401), Debio 1347 (CH5183284, S7665), FGFR4 inhibitor Roblitinib (Roblitinib-FGF401, S8548,) and pan-Src kinase inhibitor AZD0530 (S1006). All inhibitors were dissolved in dimethyl sulfoxide (DMSO), and DMSO (vehicle) was used as control in all experiments. FGFR inhibitors were generally used at a concentration of 3.6 μm (BJG398) or 5 to 10 µM (all other inhibitors), unless indicated otherwise in the text and figure legends.

### Lyn A kinase activity assay

Lyn A kinase was dissolved according to the manufacturer`s protocol for the Lyn A Kinase Enzyme System (Promega, Madison, WI; VA7476) to a final concentration of 10 ng per reaction. The enzymatic activity of Lyn A was measured in the presence of vehicle or inhibitors using the ADP-Glo™ Assay (Promega, V6930) following the manufacturer`s protocol. For each measurement, a standard curve with 100%, 60%, 20%, 5%, 3% and 0% conversion rate was made, using the provided ADP and ATP. The final concentrations in the reaction mix were 10 µM ATP and 0.2 µg/µl poly (Glu_4_, Tyr_1_) substrate. The reaction was performed for 1 h at room temperature (RT), then ADPGlo reagent was added for 30 min at RT, and the kinase reaction buffer was added for 40 min, followed by luminescence detection.

### Isolation of RNA and qRT-PCR

Total RNA was isolated from cultured cells using the IBI Scientific RNA isolation kit (IBI Scientific, Dubuque, IO; IB47303) following the manufacturer`s protocol. cDNA was synthesized using the iScript kit (Bio-Rad Laboratories, Berkeley, CA). Relative gene expression was determined using the LightCycler 480 SYBR Green system (Roche, Rotkreuz, Switzerland). Expression of the gene encoding ribosomal protein lateral stalk subunit P0 (*RPLP0*) was used for normalization of expression levels. The following primers were used:

#### RPLP0

5’-CCA CAT TGT CTG CTC CCA CA-3’ and 5’-GAA GAC AGG GCG ACC TGG AA-3’.

#### RSAD2

5’-GGA GGT GGT GCA GGG ATT AC-3’ and 5’-GGA AAA CCT TCC AGC GCA CA-3’.

#### ISG15

5’-CTT TGC CAG TAC AGG AGC T-3’ and 5’-GAC ACC TGG AAT TCG TTG C-3’.

#### ANXA2

5’-TGT GCA AGC TCA GCT TGG A- 3’and 5’-AGG TGT CTT CAA TAG GCC CAA- 3’.

#### STAT1

5’-AAA GGA AGC ACC AGA GCC AAT- 3’ and 5’-TCC GAG ACA CCT CGT CAA AC- 3.

#### IFIT1

5’-ATT TAC AGC AAC CAT GAG GAA AG- 3’ and 5’-GCT CCA GAC TAT CCT TGA CCT G- 3’.

#### NECT1

5’-CTA CCA CAT GGA CCG CTT CAA G- 3’ and 5’-CTT TGC AGG TGA GCT TCA CGT C- 3’.

#### IFNB1

5’-TGG GAG GAT TCT GCA TTA CC- 3’ and 5’-CAG CAT CTG CTG GTT GAA GA- 3’.

#### MXB

5’-TCT GTC ACT ATC AGT GTC CAT CTC TAC- 3’ and 5’-TCT TTG CTT TAT TAA ATT CCT CTT CAA- 3’.

### RNA-Seq and data analysis

HaCaT cells were cultured in DMEM/10% FBS until they reached 90-100% confluency, and treated for 5 h with DMSO, 10 µM AZD4547 or 3.6 µM BGJ398 in DMEM/5% FBS. Total RNA was isolated as described above. RNA quality and concentration were checked using a NanoDrop spectrophotometer. RNA-seq and data analysis were performed as previously described [29]. Pathway analysis was performed based on the significantly regulated genes (*p* ≤ 0.05, false discovery rate (FDR) ≤ 0.1 and log2FC ≥ 1) using Ingenuity Pathway Analysis (IPA) software, Version 26,127,183 (Qiagen, Hilden, Germany) and the built-in right-tailed Fisher Exact Test with Benjamini Hochberg (BH) multiple testing correction.

### Viral cell association assay

HaCaT cells were cultured in DMEM/10% FBS and seeded on cover slips. Cells were incubated with 4.7 × 10^5^ FFU/ml WT HSV-1 (F) in RPMI medium with 20 mM HEPES and 0.2% bovine serum albumin (BSA) for 1 h on ice to allow for viral binding, but not entry. Cells were then washed twice with cold RPMI, and the medium was replaced with DMEM/5% FBS containing vehicle (DMSO) or inhibitors. Cells were incubated for 1 h at 37 °C to allow viral entry, fixed with 4% paraformaldehyde (PFA) and stained for HSV-1 with a rabbit anti-heavy chain polyclonal antibody against purified DNA-containing HSV-1 capsids (kind gift by R. Eisenberg and G. Cohen, University of Pennsylvania, Philadelphia, PA). Cells were counterstained with DAPI and Alexa Fluor 647 NHS ester (Thermo Fisher Scientific, Waltham, MA; A200006).

After mounting coverslips on slides, the samples were imaged with a Leica SP8 inverse FALCON confocal laser scanning microscope using a 63x magnification oil objective. Signal was quantified using CellProfiler version 4.2.1, and data sorting was performed with KNIME version 4.5.1. For quantification of virus signal, nuclei were segmented based on the DAPI channel, and the resulting nuclear mask was expanded or shrunk by 10 pixels. Virus signal was quantified over the expanded (at 0 min pi) or shrunk (at 60 min pi) nuclear area.

### Staining of viral particles in 96-well plates or on cover slips

After fixation, cells were treated with 25 mM NH_4_Cl for 10 min, washed with PBS, permeabilized with Triton X-100 for 5 min, and washed again. Blocking was done with 5% goat serum for 30 min at room temperature (RT), followed by overnight incubation with the primary antibody at 4 °C. The next day, after washing with PBS, cells were stained with the secondary antibody (anti-rabbit Alexa-488) for 1 h at RT. When staining for viral cell association experiments, cells were also stained with Alexa-Fluor 647 NHS ester (Thermo Fisher Scientific, Waltham, MA; A200006) during the last washing step to visualize the cell body. The following antibodies were used: mouse anti-nucleoprotein HB65 of the Influenza strain Wisconsin (kindly provided by Dr. Yohei Yamauchi, ETH Zurich, Switzerland) and a rabbit anti-heavy chain polyclonal antibody against purified DNA-containing HSV-1 capsids (kindly provided by Drs. Robert Eisenberg and Gary Cohen, University of Pennsylvania, Philadelphia, PA) [27].

### Isolation of genomic and viral DNA from HSV-1 infected cells and from cell supernatant

Genomic and viral DNA were obtained from infected cells as previously described [30]. Viral DNA from the supernatant was isolated using the same procedure, but instead of a cell lysate, a mix of 50 µl supernatant and 100 µl lysis buffer was used in the first step. Samples were used for quantitative PCR to measure HSV-1 viral load. Primers for amplification of the human β-actin (*ACTB*) DNA (5’-TAC TCC TGC TTG CTG ATC CAC-3’ and 5’TGT GTG GGG AGC TGT CAC AT-3’) and the viral glycoprotein D (*GlycD*) DNA (5’ ACGTCCGGAAACAACCCTAC-3’ and 5’- CCCAGGTTATCCTCGCTGAC-3’) were used. For quantification of viral load in the supernatant, only *GlycD* DNA was measured, because no genomic cellular DNA was detectable in the supernatant.

### EMCV viral burden analysis

Mouse intestinal organoids were harvested using cold phosphate-buffered saline (PBS) containing 0.1% BSA. The harvested organoids were centrifuged at 300 g twice for 5 min and twice for 2 min to gradually remove the matrigel and to obtain a pellet that includes only the organoids. Once the purified pellet was obtained, RNA was isolated from the organoids as described above. To obtain enough material, organoids from 2 to 3 wells were pooled. Relative viral load was assessed by qRT-PCR using the following primers:

EMCV-3D: 5`GACGCTTGAAGACGTTGTCTTCTTA-3`, 5`-CCCTACCTCACGGAATGGGGCAAAG-3` human ACTB; 5`-TACTCCTGCTTGCTGATCCAC-3`, 5`-TGTGTGGGGAGCTGTCACAT-3`.

### Infection experiments

For all experiments, cells (HaCaT, CaCo2, Huh7, HeLa cells) were cultured in DMEM/10% FBS until they reached 90–100% confluency. HPKs were cultured in Keratinocyte-SFM medium (Gibco, Carlsbad, CA) with supplements for keratinocytes (Gibco; epidermal growth factor (EGF) #10450-013, bovine pituitary extract # 13028-014) and 10% FBS. For infection with HSV-1-GFP, cells were pre-treated overnight (ON) or for 1 h with the respective concentrations of inhibitor, while control cells were treated similarly, but with DMSO (vehicle) only. No pre-treatment with the inhibitors was performed for HSV-1 infection, unless stated otherwise. Pre-treatment of the cells and infection were done in DMEM/5% FBS. Infection with HSV-1-GFP was done at the described concentrations for 1 h at 37 °C unless stated otherwise in the figure legend, then the virus was washed away with sterile PBS, and cells were incubated in the presence or absence of inhibitors at 37 °C. Cells were fixed with 4% PFA at different time points post infection. For experiments where viral burden was assessed by extraction of gDNA followed by qPCR, cells were infected with HSV-1, but the virus was not removed until the time of cell lysis. Prior to lysis, cells were washed twice with PBS. An exception were experiments designed to measure the amount of viral DNA in the supernatant in which the virus was removed 1 h after infection, and cells were incubated in DMEM/5% FBS with DMSO or inhibitors.

For HCoV-229E/GFP infection, cells were pre-treated, then infected, and further incubated in the presence of the virus unless stated otherwise. Infected cells were incubated at 33.5 °C.

For Influenza Virus infection, cells were cultured in DMEM with 1% non-essential amino acids and 1% penicillin-streptomycin (both from Sigma-Aldrich).

### Quantification of viral infection

After fixation with 4% PFA in PBS, cells were permeabilized, and nuclei were stained with 1 µg/ml DAPI in 0.5% Triton X-100 in PBS for 5 min at RT. Samples were washed with PBS and imaged in a high-throughput microscope (IXM-XL or IXMc; Molecular Devices, San Jose, CA) in widefield mode. A 4x objective was used for plaque assays and a 20x objective to observe single-round infection. For the quantification of infection with CellProfiler [31], nuclei were segmented according to the DAPI signal, and the GFP signal over the nuclear mask was measured. For plaque assays, the number and size of the plaques were determined based on the GFP signal using the Plaque2.0 software [32]. Uninfected cells were used to distinguish between background fluorescent signal and signal resulting from viral plaques. No plaques were detectable in these cells.

### Live imaging of viral infection

For live cell imaging, cells were seeded in a flat bottom black 96-well plate (Greiner Bio-One, Kremsmünster, Austria) and incubated at 37 °C/5% CO_2_. On the next day, cells were treated with inhibitors and infected as described in the figure legends. After incubation with virus, the inoculum was removed and replaced with imaging media consisting of phenol red-free DMEM (Thermo Fisher Scientific) supplemented with 1% penicillin-streptomycin (Sigma-Aldrich), 1% L-glutamine (Sigma-Aldrich), 1% non-essential amino acids (Sigma-Aldrich), and 1 mM sodium pyruvate (final concentration), 250 ng/ml Hoechst 33,342 (Sigma-Aldrich) and 5% FBS. Live imaging was performed using an IXM-C fluorescence microscope (Molecular Devices, San Jose, CA) using a 4x objective. The plate was monitored every 15–30 min for a total time of 60 to 70 h.

### Analysis of kinome data

Data from a kinome analysis of AZD4547-treated HaCaT cells [33] were analyzed with the BioNavigator v6.3.67 software and public databases (PhosphoNet, published for in vitro or in vivo experiments, or Kinexus), as described previously [34].

### Western blot analysis

Cells were harvested in Laemmli 4x lysis buffer (16 g SDS, 48 ml Tris pH 6.8, 70 ml ddH_2_O, 80 ml glycerol), 1:1 diluted with ddH_2_O prior use. For analysis of pLyn (Y397) levels, cells were lysed in NP-40 lysis buffer (150 mM NaCl, 1% NP-40, 50 mM Tris pH 8.0) with protease inhibitors (Roche, 04 693 159 001) and phosphatase inhibitors (Roche, 04 906 837 001), placed on a rotator at 4 °C for 30 min, and centrifuged for 15 min at 13,800 g. The supernatant was transferred to new Eppendorf tubes, and lysates were stored at -80 °C. Protein concentrations were measured using BCA Protein assay (Thermo Fisher Scientific). Protein samples were then run on an SDS–PAGE and transferred to nitrocellulose membranes. After blocking of unspecific binding sites with 5% BSA (Chemie Brunschwig AG, Basel, Switzerland; PANP06-1391100), membranes were incubated with the primary antibody overnight at 4 °C (see below), followed by a horseradish peroxidase (HRP)-conjugated secondary antibody for 1 h at RT (anti-rabbit or anti-mouse IgG, Sigma-Aldrich). Signals were developed using ClarityTM Western ECL Substrate (BioRad, Hercules, CA).

The following primary antibodies were used: pLyn Tyr397 (Invitrogen; MA5-38270; 1:1000 diluted), total Lyn (Invitrogen; MA5-14924; 1:1000 diluted), Annexin A2 (Invitrogen; PA5-27085; 1:1000 diluted); pAnnexin A2 Tyr24 (Invitrogen; PA5-105372; 1:1000 diluted), vinculin (Sigma-Aldrich; v4505; 1:500 diluted), phospho-p42/44 ERK1(2 (Cell Signaling, Danvers, MA; 9101; 1:1000 diluted), total ERK1/2 (Cell Signaling; 9102; 1:1000 diluted), phospho-p38 (Cell Signaling; 9211 S; 1:1000 diluted), total p38 (Cell Signaling; 9212 S; 1:1000 diluted), GAPDH (HyTest, Turku, Finland; 5G4; 1:1000 diluted), α-tubulin (Sigma Aldrich, T5168, 1:5000 diluted), histone H3 (Abcam, Cambridge, UK; ab1791; 1:2000 diluted), total EGFR (Cell Signaling; 373746: 1:1000 diluted), pEGFR-Tyr1068 (Cell Signaling; 3777; 1:1000 diluted), and HSV-1 glycoprotein D (Abcam, ab18638, diluted 1:5000).

### Lyn knock-down in HaCaT keratinocytes

HaCaT cells were used at around 50–60% confluency. They were incubated ON with a mixture of Opti-MEM medium ^®^ (Gibco), scrambled siRNA (Negative Control #1 siRNA, Thermo Fisher Scientific, 4390843) or a mix of two Lyn siRNAs (5’-GCG ACA UGA UUA AAC AUU AUU TT-3’ and 5’-GUG AUG UUA UUA AGC ACU AUU TT-3’) and Lipofectamine™ RNAiMAX (Invitrogen; 56532). The following morning, the medium was replaced by DMEM/10% FBS. For infection experiments, cells were infected 48–60 h post transfection.

### Statistics

Statistical analysis was performed using the PRISM software, version 9 for Mac OS X or Windows (GraphPad Software Inc., La Jolla, CA). For comparison of two groups, an unpaired Student`s t test was performed; for comparison of more than two groups, Bonferroni multiple comparisons test was used.

### Supplemental information

Supplemental Information: Fig. S1-S4 and legends, Table S1.

**Fig. 1:**
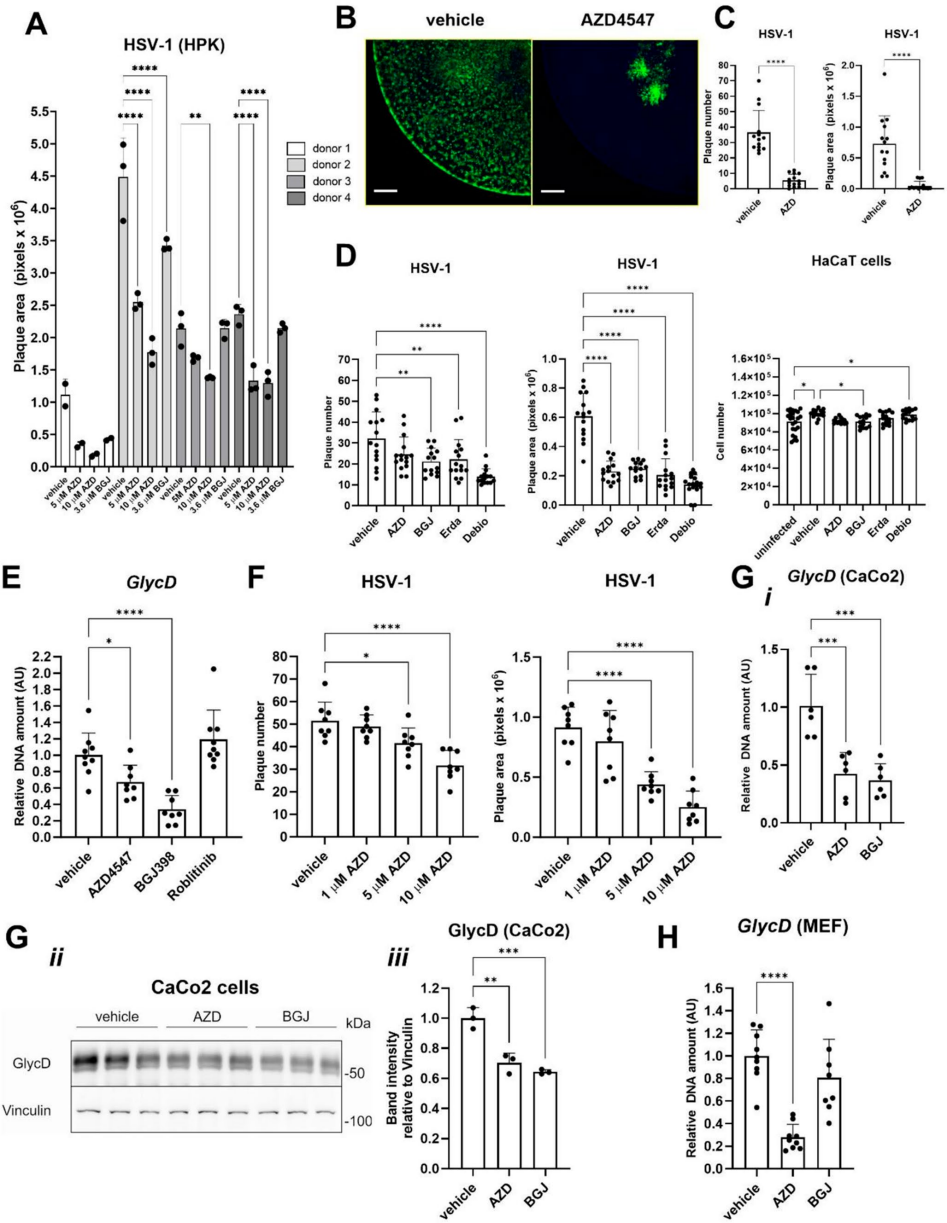
FGFR inhibitors suppress HSV-1 infection in different cell types. **A**: Area covered by viral plaques (plaque area) at 24 h post infection (hpi) of human primary foreskin keratinocytes (HPK) from four donors, infected with HSV-1-GFP (3 × 10^2^ focus-forming units (FFU)/ml) for 1 h in the presence of 5 or 10 µM AZD4547 (AZD) or BGJ398 (BGJ; 3.6 µM) or vehicle (DMSO). **B**: Live cell imaging of HaCaT keratinocytes, which had been pre-treated overnight (ON) with AZD4547 (10 µM) or vehicle and infected with HSV-1-GFP (1.5 × 10^3^ FFU/ml) for 1 h. Representative image shows the endpoint at 70 hpi, supplementary videos S1 and S2 show infection progression between 17 and 70 hpi. Magnification bar: 500 μm. **C**: Plaque number and area at 24 hpi of HaCaT keratinocytes, which had been pre-treated ON with AZD4547 (10 µM) or vehicle, and infected for 1 h with HSV-1-GFP (1.5 × 10^3^ FFU/ml). Infection was quantified at 24 hpi. **D**: Plaque number and area as well as cell number in the whole well at 24 hpi of HaCaT keratinocytes, which had been pre-treated for 1 h with AZD4547, BGJ398, LY28744, Erdafitinib (Erda), Debio-1347 (Debio)) (5 µM each) or vehicle and infected with HSV-1-GFP (1.5 × 10^3^ FFU/ml) for 1 h. **E**: qPCR for viral *GlycD* relative to host β-actin (*ACTB*) using DNA from HaCaT keratinocytes, which had been infected with HSV-1 (1.8 × 10^5^ plaque-forming units (PFU)/ml) overnight in the presence of AZD4547, BGJ398, or Roblitinib (10 µM each) or vehicle. The expression of *GlycD* was normalized to host β-actin. AU: arbitraty units. **F**: Plaque number and area at 24 hpi of HaCaT keratinocytes, which had been treated for 1 h with vehicle (ctrl) or different concentrations of AZD4547 and infected with HSV-1-GFP (1.5 × 10^3^ FFU/ml) for 1 h. **G**: qPCR for *GlycD* relative to *ACTB* (i) or Western blot (ii) for GlycD and vinculin (loading control) with quantification of the GylcD/vinculin ratio (iii) using DNA or protein lysates from CaCo-2 cells, which had been infected with HSV-1 (0.45 to 1.8 × 10^5^ PFU/ml) ON in the presence of AZD4547 (10 µM), BGJ398 (3.6 µM) or vehicle (DMSO). **H**: qPCR for *GlycD* relative to *ACTB* using DNA from mouse embryonic fibroblasts (MEFs), which had been infected with HSV-1 (1.8 × 10^5^ PFU/ml) ON in the presence of AZD4547 (10 µM), BGJ398 (3.6 µM) or vehicle (DMSO). Experiments in (**B**-**G**) were performed in the presence of DMEM/5% FBS, which contains FGFs. Bar graphs show mean +/- SD. *P < 0.05, **P < 0.01, ***P < 0.001, ****P < 0.0001. **A**, **D**-**H**: One-way ANOVA, **C**: Unpaired t-test. N (biological replicates) = 2–3 from 2 experiments (**A**), *N* = 14 from 5 experiments (**C**), *N* = 14–15 from 3 experiments (**D**), *N* = 8–9 from 3 experiments (**E**), *N* = 8 from 2 experiments (**F**), *N* = 6 (qPCR) from 2 experiments or *N* = 3 (Western blot) from 1 experiment (**G**) and *N* = 8–9 from 3 experiments (**H**)

Videos S1 and 2: Live imaging of HSV-1-GFP infection of HaCaT keratinocytes, with or without AZD4547 treatment, related to Fig. 1.

## Results

### FGFR inhibitors suppress HSV-1 infection in different cell types

To determine if FGFR inhibitors suppress viral infection of primary cells, we tested the effect of AZD4547 and BGJ398 on HSV-1 infection of human primary keratinocytes (HPKs) using concentrations that were previously used in other studies with epithelial cells [18, 35–37]. AZD4547 had strong antiviral activity in cells from four different donors when used at a concentration of 10 µM. AZD4547 at a concentration of 5 µM or BGJ398 at 3.6 µM BGJ398 were less efficient, but still showed clear effects in cells from 3 or 2 donors, respectively (Fig. 1A).

To visualize the antiviral activity of AZD4547, HaCaT keratinocytes were infected with a GFP-expressing HSV-1, and infection was live imaged for 70 h. All vehicle-treated cells were infected, and a strong cytopathic effect was observed. By contrast, only a few isolated plaques formed in the AZD4547-treated wells (Fig. 1B and Videos S1 and S2). This result shows a strong antiviral activity of AZD457, which lasts for at least 70 h post infection. Because 24 h post infection (hpi) was identified as the best time point to observe well-formed and distinct plaques suitable for quantification, we used this time point in most further experiments. There was a consistent and strong antiviral effect of AZD4547 at a concentration of 10 µM (Fig. 1C). Treatment with 5 µM of this compound, or with 5 µM BGJ398, 5 µM Erdafitinib, or 5 µM Debio-1347 also reduced the number of plaques and in particular the plaque area (Fig. 1D). No cytotoxicity was observed under these conditions (Fig. 1D, right panel). Importantly, Roblitinib, a specific inhibitor of FGFR4, a receptor that is not expressed in keratinocytes [38], did not have antiviral effects, even when used at 10 µM (Fig. 1E), as assessed by determining the amount of viral glycoprotein D (*GlycD*) DNA in the cells. Notably, viral DNA levels strictly correlated with plaque formation in all experiments, thus presenting a reliable and particularly sensitive method to quantify viral infection. The strongest effect on infection susceptibility was observed with 10 µM of AZD4547 or BGJ398, while 1 µM had only a mild or no effect (Fig. 1F and S1A). The antiviral effect of Erdafitinib and Debio-1347 was also dose-dependent (Fig. S1B), but a higher concentration of these compounds was cytotoxic. However, 10 µM AZD4547 did not affect cell viability as assessed by the number of cells at the endpoint of each infection experiment (Fig. 1D, right panel) and by measurement of the cytoplasmic enzyme lactate dehydrogenase (LDH) in the cell supernatant (Fig. S1C).

AZD4547 and BGJ398 did not affect keratinocyte proliferation as determined by Ki67 staining (Fig. S1D). Furthermore, treatment with mitomycin C, which inhibits cell proliferation, did not affect the susceptibility of HaCaT cells to HSV-1-GFP infection and did not interfere with the antiviral activity of AZD4547 (Fig. S1E), suggesting that cell proliferation is not a major determinant in our infection model. Overnight (ON) pre-treatment of cells did not significantly increase the anti-viral effects of the drugs compared to a 1 h pre-treatment (Fig. S1F), suggesting that it is not a consequence of major alterations in gene expression prior to infection. Based on this finding, all further experiments were performed with a 1 h pre-treatment, followed by continuous presence of the inhibitors in the medium, unless stated otherwise.

To exclude the possibility that the inhibitors affect GFP expression rather than the viral infection itself, we infected HaCaT cells with two native HSV-1 strains, treated the cells with FGFR inhibitors, and assessed infection at 24 hpi by immunostaining with a polyclonal antibody against HSV-1 capsid proteins [27]. We observed similar inhibitory effects of AZD4547 and BGJ398 on infection with these wild-type HSV-1 strains as we did with HSV-1-GFP (Fig. S1G).

Finally, we found that the antiviral effect of FGFR inhibition is not limited to keratinocytes, because AZD4547 and BGJ398 had strong antiviral activity against HSV-1 in CaCo2 cells, an intestinal epithelial cell line (Fig. 1G-I, i-iii). AZD4547 also suppressed HSV-1 infection of mouse embryonic fibroblasts (MEF) (Fig. 1H).

### The FGFR kinase inhibitors AZD4547 and BGJ398 suppress infection with different RNA viruses

**Fig. 2:**
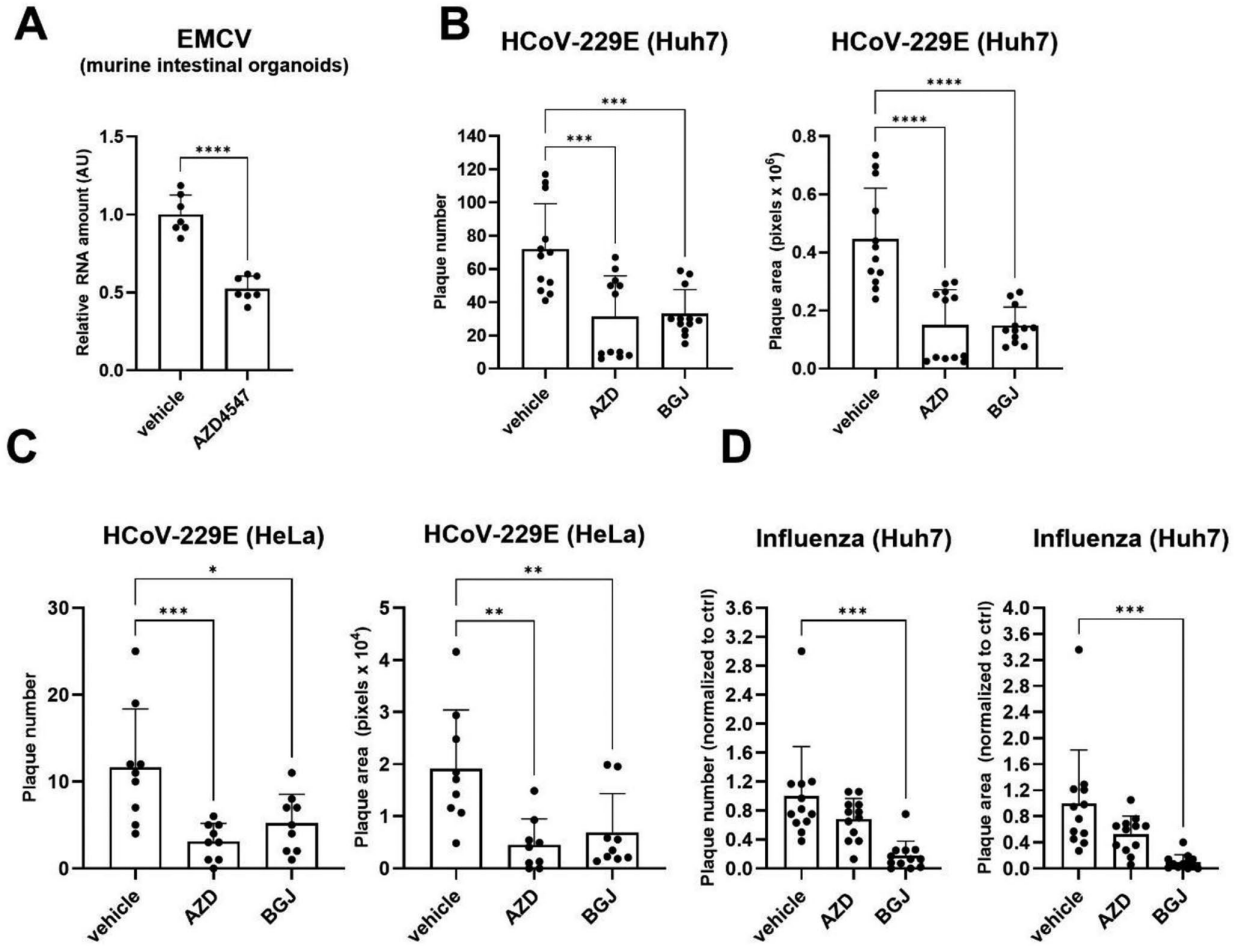
AZD4547 and BGJ398 suppress infection with different RNA viruses. **A**: qRT-PCR for the RNA encoding ECMV 3D nonstructural protein relative to host ACTB mRNA using RNA from mouse intestinal organoids, which had been cultured in full growth medium and pre-treated with AZD4547 (10 µM) or vehicle for 6 h and infected with ECMV (2.5 × 10^5^ PFU/ml). **B**: Plaque number and area at 48 hpi of Huh7 cells, which had been pre-treated with AZD4547 (10 µM), BGJ398 (3.6 µM) or vehicle for 1 h and infected with HCoV-229E-GFP (3.9 × 10^3^ FFU/ml). **C**: Plaque number and area at 48 hpi of HeLa cells, which had been pre-treated with AZD4547 (5 µM), BGJ398 (3.6 µM) or vehicle for 1 h and infected with HCoV-229E-GFP (6.3 × 10^4^ FFU/ml). **D**: Plaque number and area at 48 hpi of Huh7 cells, which had been infected with Influenza strain Wisconsin (2.05 × 10^4^ PFU/ml) in Influenza infection medium for 2 h in the presence of AZD4547 (10 µM), BGJ398 (3.6 µM) or vehicle. After removal of the virus, cells were incubated in DMEM/5% FBS including AZD4547, BGJ398 or vehicle. Bar graphs show mean +/- SD. *P < 0.05, **P < 0.01, ***P < 0.001, ****P < 0.0001. **A**: Unpaired t-test, **B**-**D**: One-way ANOVA. N (biological replicates) = 7 from 2 experiments (**A**), *N* = 12 from 4 experiments (**B**), *N* = 9 from 3 experiments (**C**) and *N* = 12 from 3 experiments (**D**)

To explore whether FGFR inhibitors modulate infection with RNA viruses, mouse intestinal organoids were infected with encephalomyocarditis virus (EMCV), a small non-enveloped single-stranded RNA virus, in the presence of AZD4547 or vehicle. A strong reduction in the amount of viral RNA was observed upon AZD4547 treatment in the absence of visible toxicity (Fig. 2A). To study the effect of the inhibitors on Corona viruses, we used Huh7 and HeLa cells, which have been routinely used in Coronavirus research [39–41]. Treatment of these cells with AZD4547 or BGJ398 significantly reduced their infection with Human Coronavirus 229E (HCov229E), an enveloped positive-sense, single-stranded RNA virus (Fig. 2B, C). Huh7 cells are also efficiently infected with Influenza Virus [42]. Their susceptibility to infection with this enveloped negative-sense RNA virus was significantly reduced by BGJ398, but only slightly by AZD4547 (Fig. 2D), suggesting that the two different inhibitors have different antiviral potential depending on the cell type and/or the virus. Table S1 summarizes the effect of AZD4547 and BGJ398 on infection of different cell types with different viruses and the bioactive inhibitor concentrations in these settings.

### AZD4547 and BGJ398 block early stages of viral infection, but not viral cell association

**Fig. 3:**
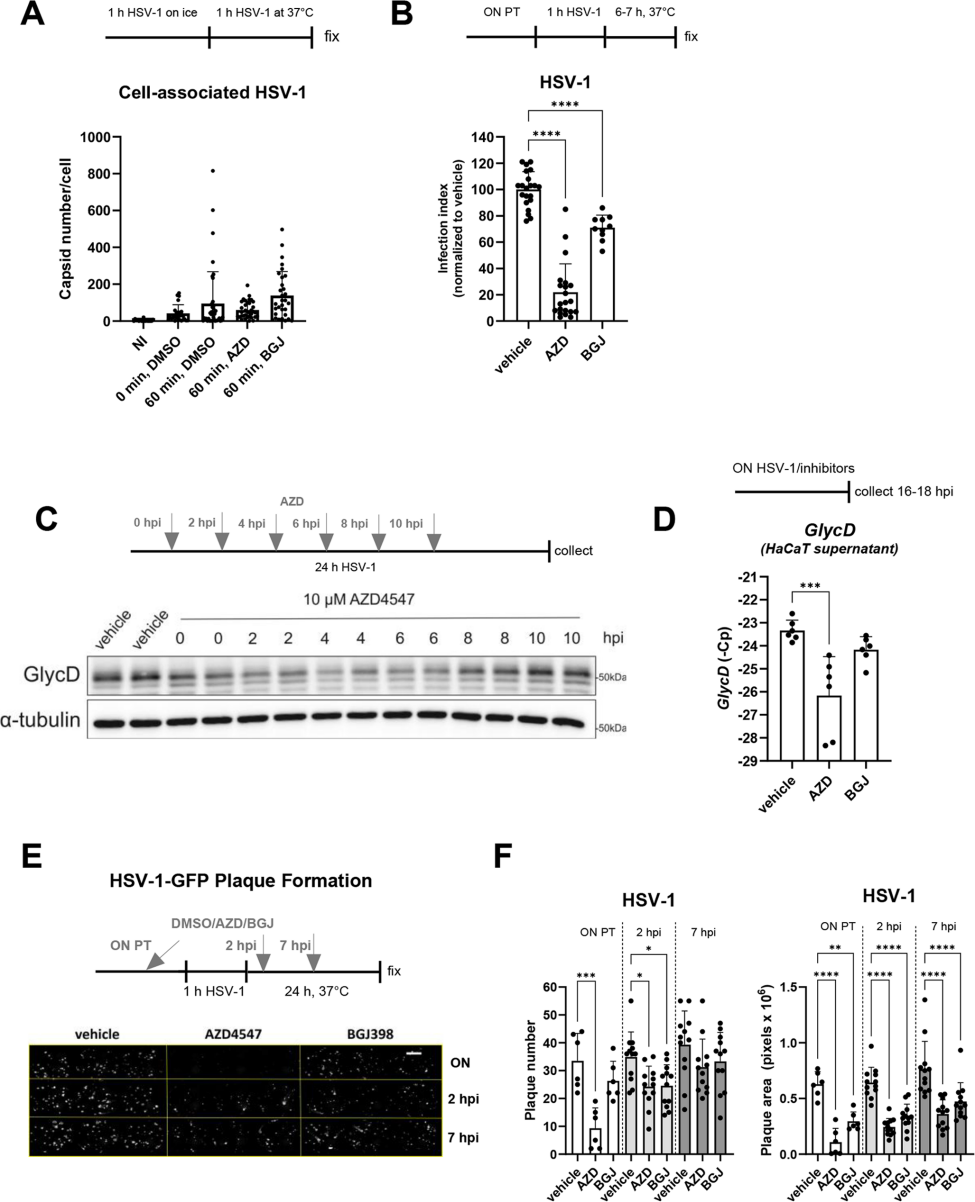
FGFR inhibitors inhibit early steps of viral infection without affecting viral entry. **A**: Number of viral capsids per cell at 1 hpi of HaCaT keratinocytes, which had been infected with WT HSV-1 on ice to allow viral attachment, washed, and cultured in fresh medium containing AZD4547 (10 µM), BGJ398 (3.6 µM) or vehicle for 1 h at 37 °C to allow firm association of the virus with the cell and viral entry. Viral particles bound to the membrane or inside the cells were identified by confocal microscopy. **B**: Infection index (number of infected cells with detectable GFP signal above the uninfected control threshold divided by the total number of cells) at 6–7 hpi of HaCaT keratinocytes, which had been pre-treated ON with AZD4547 (10 µM), BGJ398 (3.6 µM) or vehicle and infected with HSV-1-GFP (7.3 × 10^5^ FFU/ml). The infection index in the vehicle-treated cells was used for normalization within each experiment. All normalized data were then combined. **C**: Western blot analysis for GlycD and α-tubulin (loading control) using lysates from HaCaT keratinocytes, which had been infected for 24 h with HSV-1 (9 × 10^4^ PFU/ml). 10 µM AZD4547 was added at the time of infection or at different time points post infection. Vehicle was added at the time of infection. **D**: qPCR for *GlycD* using DNA from the supernatant of HaCaT keratinocytes, which had been infected ON with HSV-1 (1.8 × 10^5^ PFU/ml) in the presence of AZD4547 (10 µM), BGJ398 (3.6 µM) or vehicle. Raw Cp values are shown, because only viral DNA is present in the supernatant. **E**, **F**: Representative image of an infected plate (**E**) and graphs (**F**) showing plaque number and area at 24 hpi of HaCaT keratinocytes, which had been pre-treated ON with AZD4547 (10 µM) or BGJ398 (3.6 µM) or vehicle and infected with HSV-1-GFP (1.5 × 10^3^ FFU/ml). Alternatively, the inhibitor was added at 2–7 h post infection. Magnification bar: 3000 μm. Bar graphs show mean +/- SD. *P < 0.05, **P < 0.01, ***P < 0.001, ****P < 0.0001. A, B, D-F: One-way. ANOVA. N (biological replicates) = 3 from 2 experiments (**A**), *N* = 10–21 from 6 experiments (**B**), *N* = 6 from 2 experiments (**D**), *N* = 6–12 from 3 experiments (**F**)

Next we studied the effect of FGFR inhibition on different stages of viral infection. To investigate whether it affects firm viral cell association, we inoculated HaCaT keratinocytes with HSV-1 on ice to allow viral attachment, and then transferred the cells to 37 °C to allow firm association of the viruses with the cell and their entry. The experiment was performed in the presence of AZD4547, BGJ398 or vehicle for 60 min, followed by cell fixation, immunostaining for a viral capsid protein and counting of the cell-associated virions. There was no significant effect of the inhibitors on HSV-1 association (Fig. 3A).

To distinguish between an effect of FGFR inhibition on early vs. late stages of viral infection, HaCaT cells were infected with HSV-1-GFP for 1 h, followed by removal of the inoculum and subsequent incubation in the presence of vehicle or different FGFR inhibitors. The infection was assessed at 7 hpi, at which stage only single infected cells were visible, and no plaques had formed, thus reflecting early viral infection. Treatment with either AZD4547 or BGJ398 significantly reduced the number of cells with detectable infection at 7 hpi (Fig. 3B). Additionally, a short-term (2 h) treatment with AZD4547 was sufficient to reduce plaque area measured 24 h later in HSV-GFP infected HaCaT cells (Fig. S1H), again suggesting that the inhibitor affects the early stages of viral infection. In another experiment, we observed that the reduction of GlycD was most efficient when AZD4547 was added during the first 6 h after addition of the virus (Fig. 3C). Together, these results suggest that FGFR inhibitors do not affect HSV-1 particle association with cells, but subsequent early steps of the viral life cycle and possibly viral replication. This may reduce the number of viruses that are released from infected cells, as suggested by the strong reduction of the amount of viral DNA in the supernatant when cells were treated with AZD4547 (Fig. 3D).

Finally, we investigated if the FGFR inhibitors have post-exposure antiviral activity against HSV-1, which would be therapeutically highly relevant. HaCaT cells were infected with HSV-1-GFP, and AZD4547 and BGJ398 were added at 2 hpi or 7 hpi. At 24 hpi, both treatment regimens had significantly reduced the number of plaques and the plaque area compared to vehicle-treated cells. However, the effect was less pronounced compared to cells which had been treated with the inhibitors prior to infection (Fig. 3E, F).

### The antiviral effect of AZD4547 is independent of ISG regulation and FGFR signaling

**Fig. 4:**
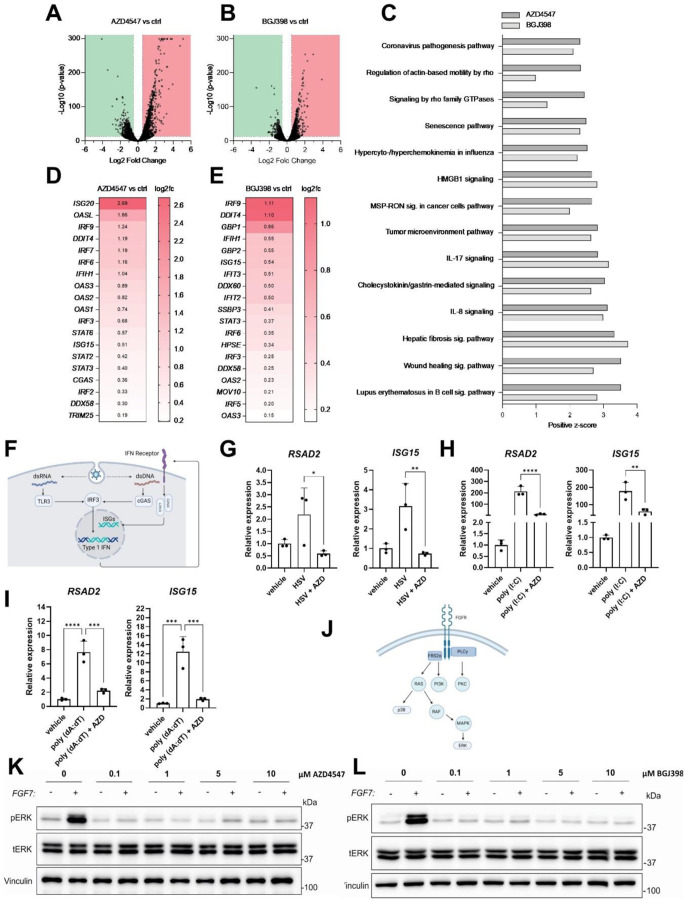
The antiviral effect of AZD4547 involves FGFR-independent pathways. **A**,** B**: Volcano plots showing RNA-seq results from HaCaT cells treated for 5 h with 10 µM AZD4547 (**A**) or 3.6 µM BGJ398 (**B**). Data points show differentially expressed genes (*p* ≤ 0.05 and FDR ≤ 0.05). **C**: Top 14 activated pathways based on differentially expressed genes (*p* ≤ 0.05, FDR ≤ 0.1 and log2FC ≥ 1) of 10 µM AZD4547- or 3.6 µM BGJ398-treated HaCaT cells compared to vehicle-treated cells according to Ingenuity Pathway Analysis (IPA). **D**, **E**: Significantly (*p* ≤ 0.05 and FDR ≤ 0.05) up-regulated ISGs based on RNA-seq data of HaCaT cells treated for 5 h with AZD4547 (**D**) or BGJ398 (**E**) compared to control. **F**: Schematic representation of interferon (IFN) signaling: In response to double-stranded RNA (dsRNA), which is sensed by toll-like receptor 3 (TLR3), or dsDNA, which is sensed by cyclic GMP-AMP synthase (cGAS), interferon-regulatory factor 3 (IRF3) gets activated, leading to expression of type I interferons. The latter induce the expression of interferon-stimulated genes (ISGs). Created with BioRender.com. **G**: qRT-PCR for *RSAD2* and *ISG15* relative to *RLPL0* using RNA from HaCaT keratinocytes, which had been pre-treated for 1 h with AZD4547 (10 µM) or vehicle and infected with HSV-1 (1.8 × 10^5^ PFU/ml) for 5 h. **H**: qRT-PCR for *RSAD2* and *ISG15* relative to *RLPL0* using RNA from HaCaT keratinocytes, which had been pre-treated ON with AZD4547 (10 µM) and incubated with poly(I:C) (2 µg/ml) for 3 h. **I**: qRT-PCR for *RSAD2* and *ISG15* relative to *RLPL0* using RNA from HaCaT keratinocytes, which had been pre-treated for 1 h with AZD4547 (10 µM) and incubated with poly(dA:dT) (1 µg/ml) for 6 h. **J**: Schematic representation of canonical FGFR signaling: Upon ligand binding and dimerization, FGFR signaling activates different intracellular pathways, including the RAS-ERK1/2, RAS-RAC-p38, PI3K (phosphoinositide-3 kinase), PLCγ (phospholipase C γ) pathways. Created with BioRender.com. **K**: Western blot analysis for phosphorylated and total ERK and vinculin using lysates from HaCaT keratinocytes, which had been serum-starved overnight, treated for 1 h with different concentrations of AZD4547 or vehicle and incubated for 15 min with FGF7 (10 ng/ml). **L**: Western blot analysis for phosphorylated and total ERK and vinculin using lysates from HaCaT keratinocytes, which had been serum-starved overnight, treated for 1 h with different concentrations of BGJ398 or vehicle and incubated for 15 min with FGF7 (10 ng/ml). Bar graphs show mean +/- SD. *P < 0.05, **P < 0.01, ****P < 0.0001. G-I: One-way ANOVA. N (biological replicates) = 3 from one experiment (**G**-**I**)

We previously showed that FGFs suppress the interferon response and that the promotion of viral infection by FGF7 correlates with reduced expression of ISGs in HaCaT keratinocytes [7]. Therefore, we hypothesized that the antiviral effect of FGFR inhibitors could be mediated, at least in part, by upregulation of ISGs. To address this question, we performed RNA sequencing (RNA-seq) of HaCaT cells treated with vehicle, or 10 µM AZD4547 or 3.6µM BGJ398 for 5 h. Among several thousand genes that were regulated by FGFR inhibitor treatment (Fig. 4A, B), expression of 642 genes was upregulated by both inhibitors (*p* ≤ 0.05, FDR ≤ 0.05, and log2FC ≥ 0.5), and expression of 679 genes was downregulated by both inhibitors (*p* ≤ 0.05, FDR ≤ 0.05, and log2FC ≤ -0.5).

Ingenuity Pathway Analysis (IPA) uncovered a significant enrichment of pathways associated with infectious diseases and cytokine signaling (Fig. 4C), indicating a pronounced induction of a pro-inflammatory and antiviral state following pharmacological FGFR inhibition. A panel of ISGs was indeed significantly upregulated upon AZD4547 or BGJ398 treatment (Fig. 4D, E). In virus-infected cells, viral double-stranded RNA or DNA induce type I interferons, which then promote the expression of a large set of ISGs [43] (Fig. 4F). However, when HaCaT cells were treated with AZD4547 and infected with HSV-1, expression of two classical ISGs - *RSAD2* and *ISG15* - was suppressed rather than enhanced (Fig. 4G). Because AZD4547 treatment reduced the viral load and because HSV-1 infection increases ISG expression [43], we tested if AZD4547 affects ISG expression of HaCaT cells treated with poly(I:C), a double-stranded RNA viral mimetic [44], whose effect on ISG expression was shown to be suppressed by FGFR signaling [7]. To mimic the effect of the DNA virus HSV-1, we used poly(dA:dT), a double-stranded DNA mimetic. Akin to HSV-1 infection, AZD4547 treatment decreased ISG expression in response to poly(I:C) or poly(dA:dT) treatment, suggesting that the anti-viral effect of AZD4547 does not depend on ISGs induction (Fig. 4H, I). This was reinforced by analysis of multiple time points and different experimental settings (Fig. S2A-C).

Finally, we tested if the effect of AZD4547 on viral infection is a consequence of FGFR kinase inhibition by studying the effect of the inhibitors on the phosphorylation (activation) of ERK1/2 and p38, which are key components of the FGF signaling pathway (Fig. 4J). Remarkably, higher concentrations of the FGFR inhibitors were required for inhibition of viral replication than for inhibition of FGF7-induced ERK1/2 and p38 activation. While ERK1/2 and p38 activation was blocked with 0.1 µM AZD4547 and 0.1 µM BGJ398 (Fig. 4K, L, and S2D), viral inhibition required a concentration of at least 1 µM (see Fig. 1F).

### AZD4547 inhibits Src kinases

**Fig. 5:**
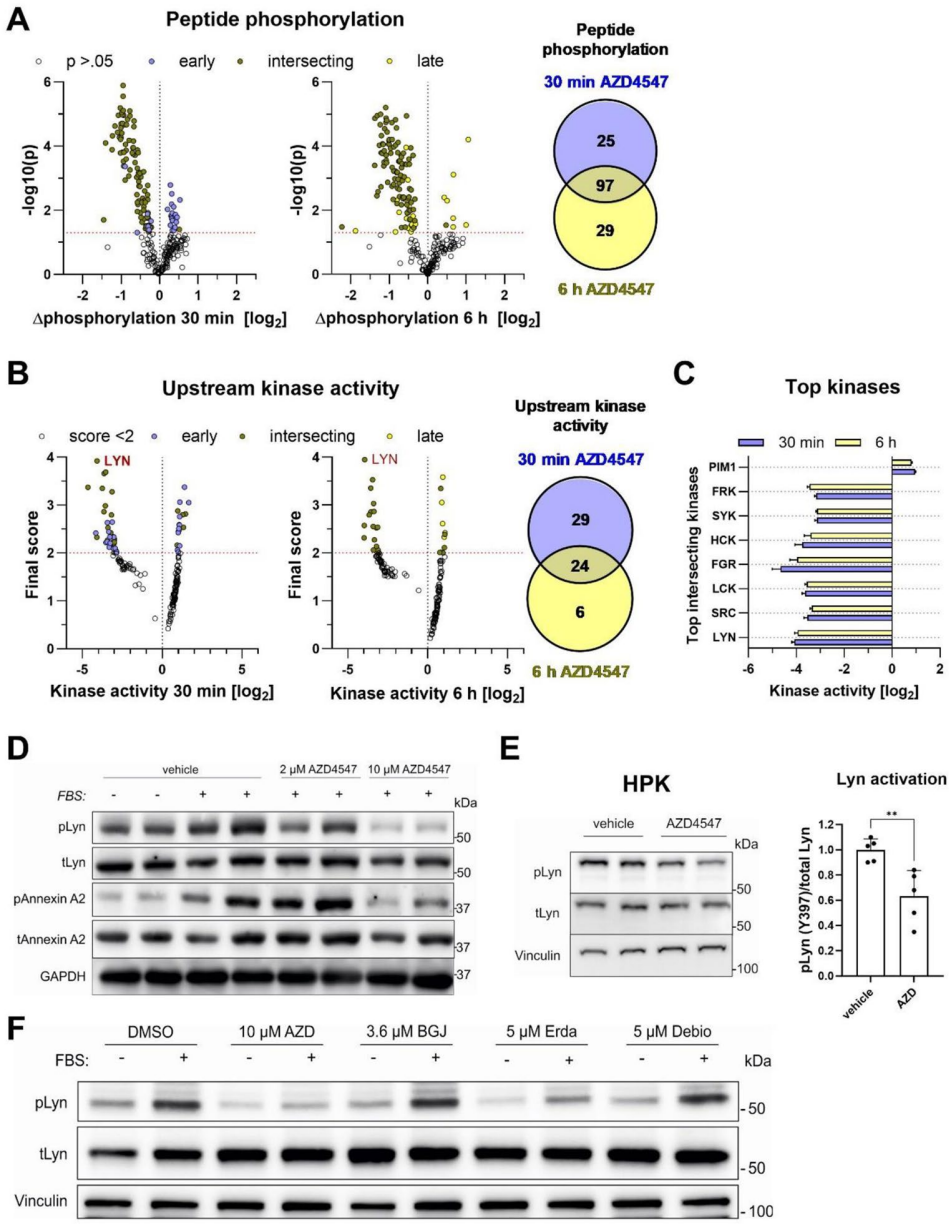
Kinome profiling identifies Src family kinase members as targets of AZD4547. (**A-C**) Analysis of kinome data from HaCaT cells, which had been treated with AZD4547 (10 µM) or vehicle for 30 min or 6 h [33], for phosphorylation of distinct immobilized peptides and prediction of responsible upstream kinases. **A**: Volcano plots showing differential peptide phosphorylation in log2 space (x-axis) correlated with its significance expressed as–log10[p-value] (y-axis); data points represent single peptides; thresholding (red dashed line) applied at *p* > 0.05; x < 0 indicates lower phosphorylation upon treatment; x > 0 indicates higher phosphorylation upon treatment; Venn-diagram depicts number of significantly differentially phosphorylated peptides at 30 min (blue; early), 6 h (yellow; late) or both time points (light green). **B**: Volcano plots showing predicted upstream kinase activity in log2 space (x-axis) correlated with its final score (combinatory score including significance, specificity and extent of deregulation, y-axis) based on phosphorylation-pattern in (A); data points represent single kinases; thresholding (red dashed line) applied at score < 2; x < 0 indicates lower kinase activity upon treatment; x > 0 indicates higher kinase activity upon treatment; Venn-diagram depicts number of highly scored kinases solely at 30 min (blue; early), 6 h (yellow; late) or both time points (light green). **C**: Depiction of differential kinase activity of top hit kinases in log2 space. **D**: Western blot analysis for phosphorylated and total Lyn and phosphorylated and total Annexin A2 and GAPDH using lysates from HaCaT keratinocytes, which had been serum-starved ON, pre-treated with AZD4547 (10 µM) or vehicle for 1 h and incubated with 5% FBS for 15 min. **E**: Western blot analysis for phosphorylated and total Lyn and vinculin using lysates from HPKs, which had been treated with 10 AZD4547 (µM) or DMSO for 1 h. Bar graph shows pLyn/total Lyn ratio. **F**: Western blot analysis for phosphorylated and total Lyn and vinculin using lysates from HaCaT keratinocytes, which had been serum-starved ON, pre-treated for 1 h with AZD4547 (10 µM), BGJ398 (3.6 µM), Erdafitinib (5 µM), Debio-1347 (5 µM) or vehicle and then incubated with 5% FBS for 15 min. Bar graphs show mean +/- SD. **P < 0.01., E: Unpaired t-test. N (biological replicates) = 3 from 1 experiment (**A**-**C**), and *N* = 5 from 2 experiments (**E**) biological replicates

To identify potential off-targets of FGFR kinase inhibitors that could be relevant for their anti-viral activity, we re-analyzed the data of a recently performed kinome study of AZD4547-treated HaCaT cells [33]. This unbiased approach provides information on kinases, whose activity is modulated upon a particular treatment. In that study, HaCaT cells had been treated with 10 µM AZD4547 or vehicle for 30 min or 6 h, and kinase activities were analyzed at both time points. The phosphorylation of 97 peptides was affected at both time points, while other peptides were only altered at one time point (Fig. 5A). Analysis of the kinases, which are able to phosphorylate these peptides and are likely to be inhibited by AZD4547, identified Src family kinase (SFK) members as major targets at both time points. Lyn kinase was the top hit in terms of significance (Fig. 5B) and among the proteins with a particularly strong fold-change in activity (Fig. 5C). Although most kinases were inhibited by AZD4547, a few were even activated, including Pim1 (Fig. 5C; [33]). Because Lyn kinase was among the top hits and because SFKs participate in multiple cellular processes, including viral infections [45], we focused our follow-up experiments on SFKs.

Src has been shown to get phosphorylated at tyrosine residues in response to FGF7 treatment of keratinocytes, resulting in its activation [46]. This activation step occurs most likely via direct phosphorylation by the FGFR kinase. However, in our settings, Src phosphorylation was not affected by AZD4547 or BGJ398 (Fig. S3A), whereas Lyn activation was reduced by treatment with 10 µM or 5 µM, but not 2 µM, 1 µM or 100 nM AZD4547, as assessed by its phosphorylation at Tyr397, which activates Lyn kinase [47] (Fig. 5D, Fig. S3B). The concentrations required for the inhibition of Lyn correlate with those required for the antiviral activity of AZD4547 (Fig. 1F). Phosphorylation of annexin A2, a downstream target of Lyn [48], was similarly reduced upon treatment with 10 µM AZD4547 (Fig. 5D). AZD4547 treatment (10 µM) also suppressed Lyn phosphorylation in HPKs (Fig. 5E). Erdafitinib, but not BGJ398 or Debio-1347, had a mild inhibitory effect in this setting, but AZD4547 was the most potent Lyn inhibitor (Fig. 5F). Together, these results suggest a role of Lyn in the antiviral activity of FGFR inhibitors, in particular of AZD4547, but inhibition of additional SFK family members may also play a role.

### AZD4547 inhibits Lyn phosphorylation at Tyr397 independently of FGFR2

**Fig. 6:**
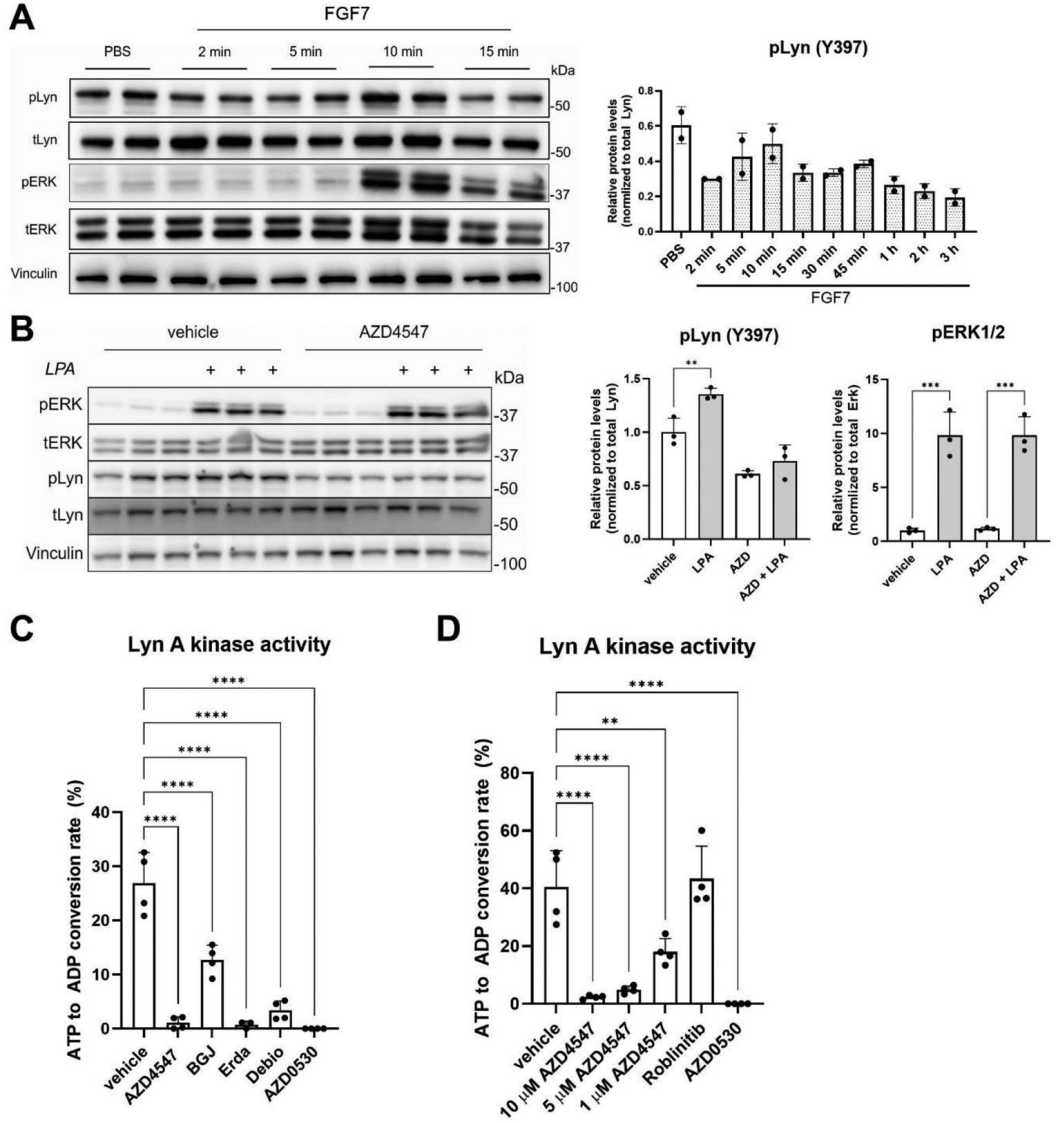
AZD4547 inhibits Lyn phosphorylation at Tyr397 independent of FGFR2 signaling. **A**: Western blot analysis for phosphorylated and total Lyn and ERK1/2 and vinculin using lysates from HaCaT keratinocytes, which had been starved ON and treated with 10 ng/ml FGF7 for 2 min to 3 h. Graph shows quantification of the pLyn/total Lyn ratio. **B**: Western blot analysis for phosphorylated and total Lyn and ERK1/2 and vinculin using lysates from HaCaT keratinocytes, which had been serum-starved ON, pre-treated for 1 h with AZD4547 (10 µM) or vehicle and incubated with 10 µM lysophosphatidic acid (LPA) for 15 min. Graphs show quantification of the pLyn/total Lyn and pERK1/2/total ERK1/2 ratios. **C**: Kinase activity of recombinant Lyn A upon incubation with AZD4547 (10 µM), BGJ398 (3.6 µM), Erdafitinib (5 µM), Debio-1347 (5 µM), AZD0530 (5 µM) or vehicle for 1 h. **D**: Kinase activity of recombinant Lyn A upon incubation with AZD4547 (1–10 µM), the FGFR4 inhibitor Roblitinib, the pan-SFK inhibitor AZD0530 (10 µM each) or vehicle for 1 h. Bar graphs show mean +/- SD. *P < 0.05, **P < 0.01, ****P < 0.0001. B-D: One-way ANOVA. N (biological replicates) = 2 from 1 experiment (**A**), *N* = 3 from 1 experiment (**B**) and *N* = 3–4 from 2 experiments (**C**, **D**)

We next determined if Lyn activation is modulated by FGFR signaling. However, no increase in Lyn phosphorylation at Y397 was observed in HaCaT cells after a 2 min–3 h treatment with FGF7 (Fig. 6A). Furthermore, the amounts of pLyn (Y397) and total Lyn at the membrane were not affected by FGF7 treatment (Fig. S3C). Therefore, we hypothesized that AZD4547 may directly inhibit the activity of Lyn.

To test this hypothesis, HaCaT cells were treated with lysophosphatidic acid (LPA), which activates Lyn through the LPA receptor (LPAR) [49], a G protein-coupled receptor. Stimulation with LPA mildly increased the levels of pLyn (Y397) (Fig. 6B). Treatment with AZD4547 suppressed the basal levels of pLyn, and it also blocked the induction of Lyn phosphorylation by LPA without blocking LPAR signaling as demonstrated by the unaltered pERK1/2 levels. These results suggest a direct effect of AZD4547 on Lyn phosphorylation. To further test this possibility, we performed a cell-free kinase assay in which purified recombinant Lyn A kinase was incubated with DMSO (vehicle), AZD4547, BGJ398, Erdafitinib, Debio-1347, or the pan-SFK inhibitor AZD0530 as positive control. Lyn A was selected for the cell-free assay, because both *LynA* and *LynB* are expressed at similar levels in HaCaT cells (Fig. S4A, two left lanes). AZD0530 completely suppressed Lyn A kinase activity. In addition, all FGFR kinase inhibitors reduced Lyn A activity in this assay (Fig. 6C), although BGJ398 and Debio-1347 had only a mild effect on Lyn phosphorylation (Y397) (Fig. 5F). It seems likely that the cell-free kinase assay is more sensitive than the Western blot analysis of phosphorylated Lyn. However, and consistent with the Western blot data, the effect of BGJ398 on Lyn activity was significantly milder compared to AZD4547, which mirrors the lower antiviral activity that BGJ398 exhibits against HSV-1 in HaCaT cells. The effect of AZD4547 on Lyn A kinase activity was dose-dependent and correlated with the antiviral activity of this compound (Figs. 1F and 6D). Remarkably, it was already visible at 1 µM AZD4547, a concentration that is frequently used in vitro and that was measured in the serum of patients treated with FGFR inhibitors [19]. By contrast, the FGFR4 inhibitor Roblitinib, which had no effect on HSV-1 infection (Fig. 1E), did not affect Lyn activity, even at 10 µM concentration (Fig. 6D).

### Lyn is required for the antiviral activity of AZD4547

**Fig. 7:**
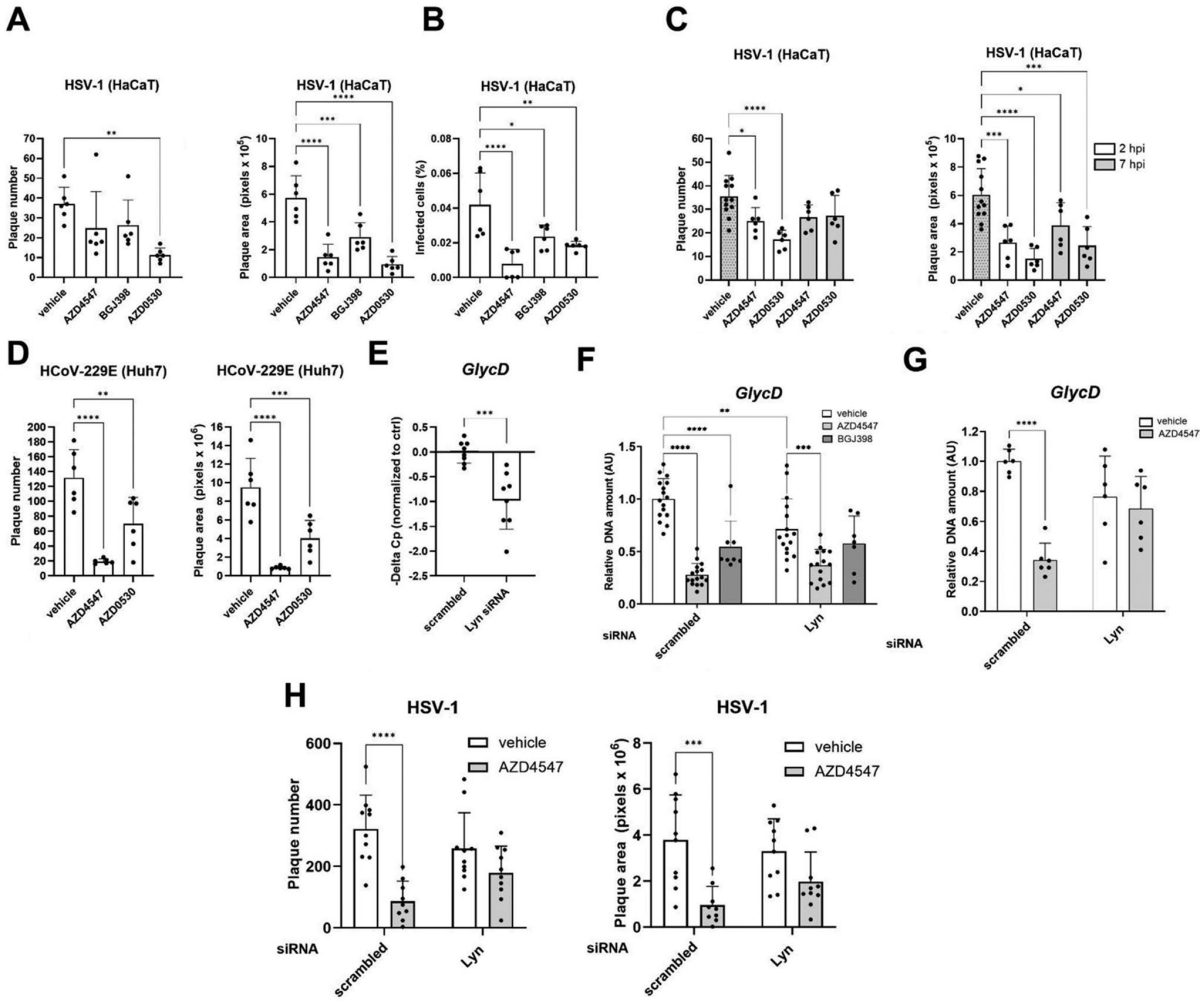
Lyn is required for the antiviral activity of AZD4547. **A**: Plaque number and area at 24 hpi of HaCaT keratinocytes, which had been pre-treated for 1 h with AZD4547 (10 µM), BGJ398 (3.6 µM), AZD0530 (5 µM) or vehicle and infected with HSV-1-GFP (1.5 × 10^3^ FFU/ml) for 1 h. **B**: Percentage of infected HaCaT keratinocytes at 7 hpi. Cells had been pre-treated for 1 h with AZD4547 (10 µM), BGJ398 (3.6 µM), AZD0530 (5 µM) or vehicle and were then infected with HSV-1-GFP (1.5 × 10^5^ FFU/ml) for 1 h. **C**: Plaque number and area at 24 hpi of HaCaT keratinocytes, which had been infected with HSV-1-GFP (1.5 × 10^3^ FFU/ml) for 1 h and treated with AZD4547 (10 µM), AZD0530 (5 µM) or vehicle at 2 or 7 hpi. **D**: Plaque number and area at 48 hpi of Huh7 cells, which had been pre-treated with AZD4547 (10 µM), AZD0530 (5 µM) or vehicle for 1 h and infected with HCoV-229E-GFP (3.9 × 10^3^ FFU/ml). **E**: Relative delta Cp change measured through qPCR for *GlycD* using DNA from the supernatant of HaCaT keratinocytes, which had been transfected with scrambled or Lyn siRNA and infected at 48–60 h post transfection with HSV-1 (1.8 × 10^5^ PFU/ml) for 1 h, followed by ON incubation in the absence of virus. Raw Cp values are used, because only viral DNA is present in the supernatant. The Cp value for the scrambled siRNA treated samples was averaged in each experiment and used to calculate the delta change in the amount of viral DNA (delta Cp = Cp average ctrl – Cp sample). **F**: qPCR for *GlycD* relative to *ACTB* using DNA from HaCaT keratinocytes, which had been transfected with scrambled (ctrl) or Lyn siRNA and infected at 48–60 h post transfection with HSV-1 (1.8 × 10^5^ PFU/ml) ON in the presence of AZD4547 (10 µM), BGJ398 (3.6 µM) or vehicle. **G**: qPCR for *GlycD* relative to *ACTB* using DNA from HaCaT keratinocytes, which had been transfected with scrambled (ctrl) or Lyn siRNA and infected at 48–60 h post transfection with HSV-1 (1.8 × 10^5^ PFU/ml) for 20 h in the presence of AZD4547 (10 µM) or vehicle. **H**: Plaque number and area at 48 hpi of HaCaT keratinocytes, which had been transfected with scrambled or Lyn siRNA, infected at 48–60 h post transfection with HSV-1-GFP (1.5 × 10^3^ FFU/ml) for 1 h and incubated in medium with AZD4547 (10 µM) or vehicle. Bar graphs show mean +/- SD. *P < 0.05, **P < 0.01, ***P < 0.001, ****P < 0.0001. A-D: One-way ANOVA, F-H: Two-way ANOVA, E: Unpaired t-test. N (biological replicates) = 6 from 2 experiments (A and B), *N* = 6–12 from 2 experiments (**C**), *N* = 6 from 2 experiments (**D**), *N* = 8 from 3 experiments (**E**), *N* = 8–16 from 7 experiments (**F**), *N* = 6 from 3 experiments (**G**), *N* = 9–10 from 3 experiments (**H**)

Finally, we studied whether Lyn plays a role in the susceptibility to infection of different cell types with different viruses. The pan-SFK inhibitor AZD0530 was first used for this purpose, because a specific Lyn inhibitor is not available. Indeed, treatment with this compound strongly reduced infection of HaCaT cells with HSV-1-GFP (Fig. 7A). Similar to AZD4547, the SFK inhibitor suppressed the early stages of infection of HaCaT cells with HSV-1-GFP (Fig. 7B), but was still efficient when added to infected cells at 2 hpi (Fig. 7C). A milder effect was seen when it was added at 7 hpi (Fig. 7C). In addition, SFK inhibition promoted resistance to infection of Huh7 cells with HCoV-229E, although to a lesser extent than AZD4547 (Fig. 7D).

To specifically determine the role of Lyn in this context, it was knocked down in HaCaT cells with individual Lyn siRNAs or a combination of two siRNAs (Fig. S4B-D). Cells transfected with scrambled siRNA were used as control. At 48–60 h post transfection, cells were infected with HSV-1, incubated ON, and the amount of viral *GlycD* DNA in the supernatant was quantified. Indeed, Lyn knock-down significantly reduced the amount of viral DNA in the supernatant (Fig. 7E). However, when the effect of Lyn knock-down on viral load of the cells was analyzed, we observed a smaller and variable effect, which was dependent on the time point studied as well as on the viral quantification method (Fig. 7F-H). This variability may result from the incomplete knock-down of Lyn. Furthermore, the kinome data suggest that additional Src kinases are inhibited by AZD4547. Consistent with both possibilities, AZD4547, but not BGJ398, still had anti-viral activity in Lyn knock-down cells (Fig. 7F), but it was much milder compared to control cells. At a later time point (20 hpi), the antiviral activity of AZD4547 was completely abolished in Lyn knock-down cells (Fig. 7G). Next, HaCaT cells were transfected with scrambled or Lyn siRNA, infected with HSV-1-GFP, and infection was measured at 48 hpi. This later time point was selected after a live imaging experiment, where the effect of the knock-down on infection was mainly visible after 40 h. At 48 hpi, the plaques in the inhibitor-treated conditions were still sufficiently distinct, allowing reliable quantification (Fig. S4E). While Lyn knock-down had no significant effect on HSV-1 plaque number and area in this setting in vehicle-treated cells, it reduced the antiviral effect of AZD4547 (Fig. 7H). Taken together, these results suggest that AZD4547 and most likely other FGFR kinase inhibitors exert their antiviral activities by inhibiting Lyn kinase and possibly additional SFKs.

## Discussion

We discovered a potent antiviral activity of several FGFR kinase inhibitors in different cell types. These compounds affect the early stages of viral infection without affecting viral cell association. Remarkably, FGFR inhibitors suppressed infection of different cell types with different RNA or DNA viruses, although with different efficiency. A recent study reported that Zika virus infection is also inhibited by AZD4547, but the effect was rather mild. The relatively poor antiviral activity was attributed to the activation of Pim1 kinase by this compound, which affected innate immunity and the endolysosomal system. This study identified Pim1 as a potential drug-target or vaccine adjuvant [33]. In the future, it will be interesting to determine if the antiviral activity of AZD4547 and potentially other FGFR inhibitors is also modulated by Pim1 in other cell types and if different viruses are differently affected by Pim1 activation. This will be relevant for the improvement of antiviral approaches.

Based on our previous results, which showed that FGFR signaling suppresses the expression of ISGs in keratinocytes [7], we initially speculated that the antiviral activity of FGFR kinase inhibitors results from increased expression of ISGs. The latter comprise a large family of genes, whose products cooperate to inhibit viral entry, protein translation and replication, as well as viral assembly and egress [43]. Although AZD4547 and BGJ398 indeed induced the expression of several ISGs in the absence of infection, there was no correlation between the expression of ISGs and the effect of the different FGFR inhibitors on susceptibility to infection in virus-infected cells, suggesting that other mechanisms are involved.

Our follow-up studies revealed that the antiviral activity of FGFR kinase inhibitors is largely independent of their effect on FGFR kinase activity. Thus, relatively high concentrations of the inhibitors (1–10 µM) were required for efficient inhibition of viral infection, although FGFR signaling was already inhibited at a much lower concentration (0.1 µM). The concentrations used in this study were originally selected based on previous in vitro studies and clinical trial data, where plasma levels of AZD4547 in cancer patients receiving long-term AZD4547 treatment were around 0.5–1 µM [19]. We speculated that a short-term treatment for viral infection would be possible with similar or even higher doses. The relatively high concentration that was required for potent antiviral activity suggested a possible off-target effect that is highly relevant for viral infection, but does not cause cytotoxicity. Indeed, analysis of kinome data from AZD4547-treated HaCaT cells [33] showed a strong effect of AZD4547 on the activity of several SFKs, with Lyn being among the top hits. We focused on validating Src and Lyn, since our RNA-seq data from HaCaT cells showed undetectable or low expression of most other SFKs in these cells. While Src activity was not obviously affected by AZD4547, Lyn activation was significantly reduced by all tested FGFR inhibitors, with AZD4547 having the strongest effect and BGJ398 having a mild effect. This is consistent with the antiviral activities of the different kinase inhibitors. Importantly, the minimal concentration of the inhibitors that was required for Lyn kinase inhibition was also the minimal concentration required for the inhibition of viral infection.

A connection between Src and FGFR signaling is well described in the literature, and some studies even showed a direct interaction between FGFR and Src [50]. However, FGF7 did not affect Lyn kinase activity in keratinocytes. By contrast, all tested compounds that inhibit FGFR1, FGFR2, and FGFR3 inhibited Lyn kinase activity in the in vitro kinase assay.

All these results showed a strong correlation between Lyn activity and susceptibility to viral infection. Consistently, a pan-SFK inhibitor had strong and broad antiviral activity, similar to the FGFR inhibitors. In addition, Lyn knock-down alone reduced or completely abolished the antiviral activity of AZD4547 in different experimental settings. These results suggest that inhibition of Lyn activity is the major mechanism underlying the activity of AZD4547, although additional Src kinases are likely to be involved. In the future it will be interesting to determine if AZD4547 and other FGFR inhibitors directly bind to Lyn and which domains are required for this interaction.

Lyn was originally identified as a hematopoietic cell-specific kinase [51], and it is mainly known for its functions in immune cells [47, 52–54]. Later, it was found to orchestrate intracellular signaling in numerous hematopoietic cell types [55, 56]. However, in spite of the important function of Src in the control of viral infections and the documented antiviral effect of pan-Src kinase inhibitors [57–60], there is little information on the role of Lyn in viral disease. A recent study demonstrated that pharmacological inhibition or genetic deletion of Lyn blocked the secretion of Dengue and Zika viral particles in different non-hematopoietic cells [61]. The reduced amounts of viral DNA that we detected in the supernatant of HSV-1-infected keratinocytes upon Lyn knock-down point to a similar function of Lyn in the control of HSV-1 particle secretion. However, the amounts of intracellular viral DNA and proteins were also reduced, suggesting additional functions of Lyn in this setting, which remain to be determined.

In conclusion, we observed a strong and robust antiviral activity of different FGFR inhibitors in vitro, which is based on an unexpected inhibition of Src family kinases, particularly of Lyn. This is of potential clinical relevance, because some of the studied FGFR inhibitors are in clinical trials for cancer [62–64]. Therefore, they may be repurposed for the treatment of viral infections. Furthermore, the role of Lyn in the susceptibility to viral infections that we discovered in this study suggests Lyn as a promising therapeutic target for the treatment of different viral infections. Finally, the effect of FGFR inhibitors on Src kinases should be taken into consideration when FGFR inhibitors are used for the treatment of cancer or other diseases associated with increased FGFR kinase activity, in particular when applied at higher concentrations.

## Electronic supplementary material

Below is the link to the electronic supplementary material.

Supplementary Material 1

Supplementary Material 2

## Data Availability

All data are shown in the Figures or Supplementary Figures. Original RNA-seq files are deposited in the Gene Expression Omnibus (GEO) (GSE267537). Any additional information required to re-analyze the data reported in this paper is available from the corresponding authors upon request.
