# Supplementary material for: FGF receptor kinase inhibitors exhibit broad antiviral activity by targeting Src family kinases
Source: Cell Mol Life Sci. 2024 Dec 2;81(1):471. doi: 10.1007/s00018-024-05502-x (PMC11612106; doi:10.1007/s00018-024-05502-x)
Supplement: Supplementary file 1 — Supplementary Material 1 [file 18_2024_5502_MOESM1_ESM.pptx]

## Slide 1
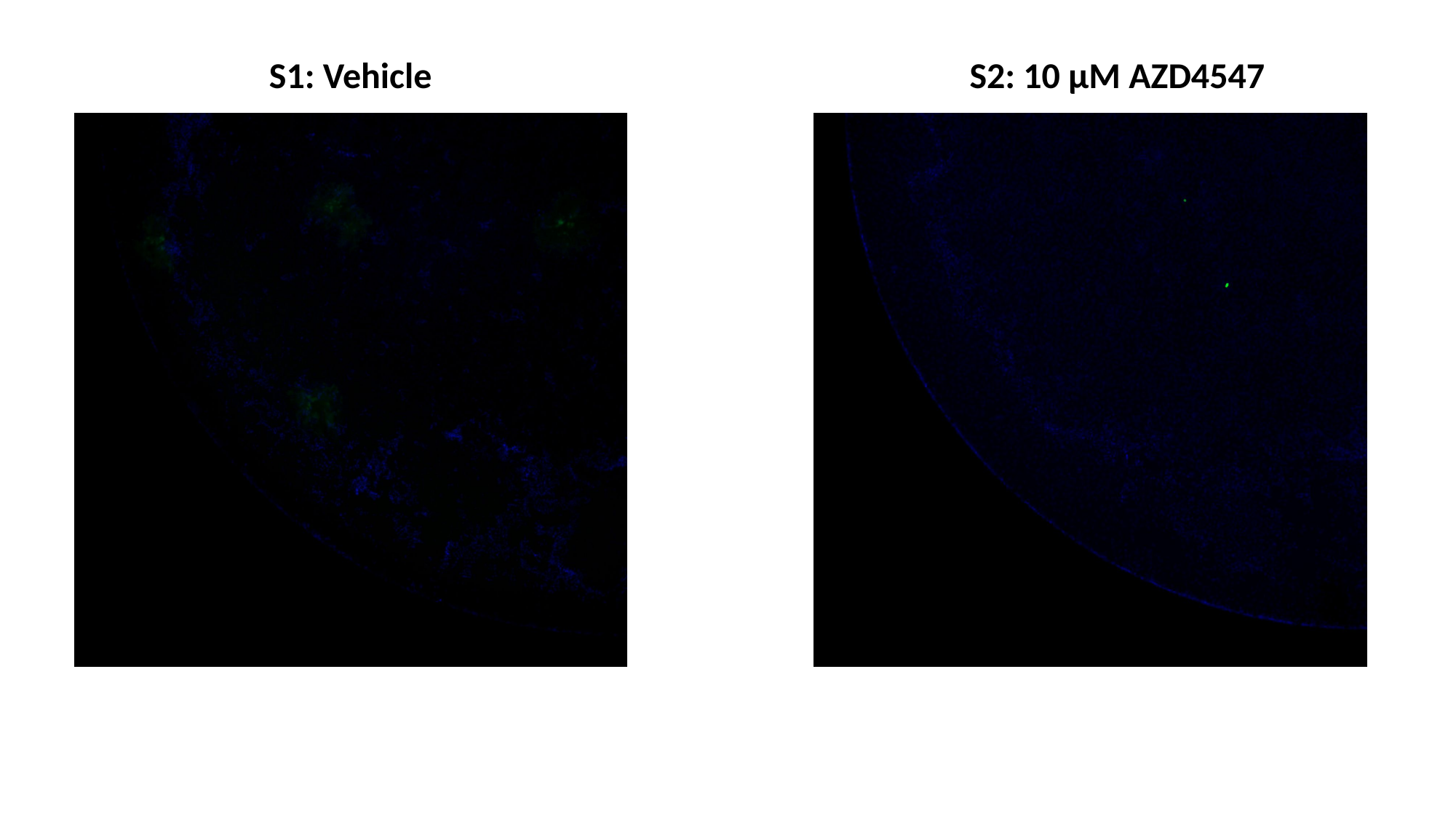

S1: Vehicle
S2: 10 µM AZD4547

## Slide 2
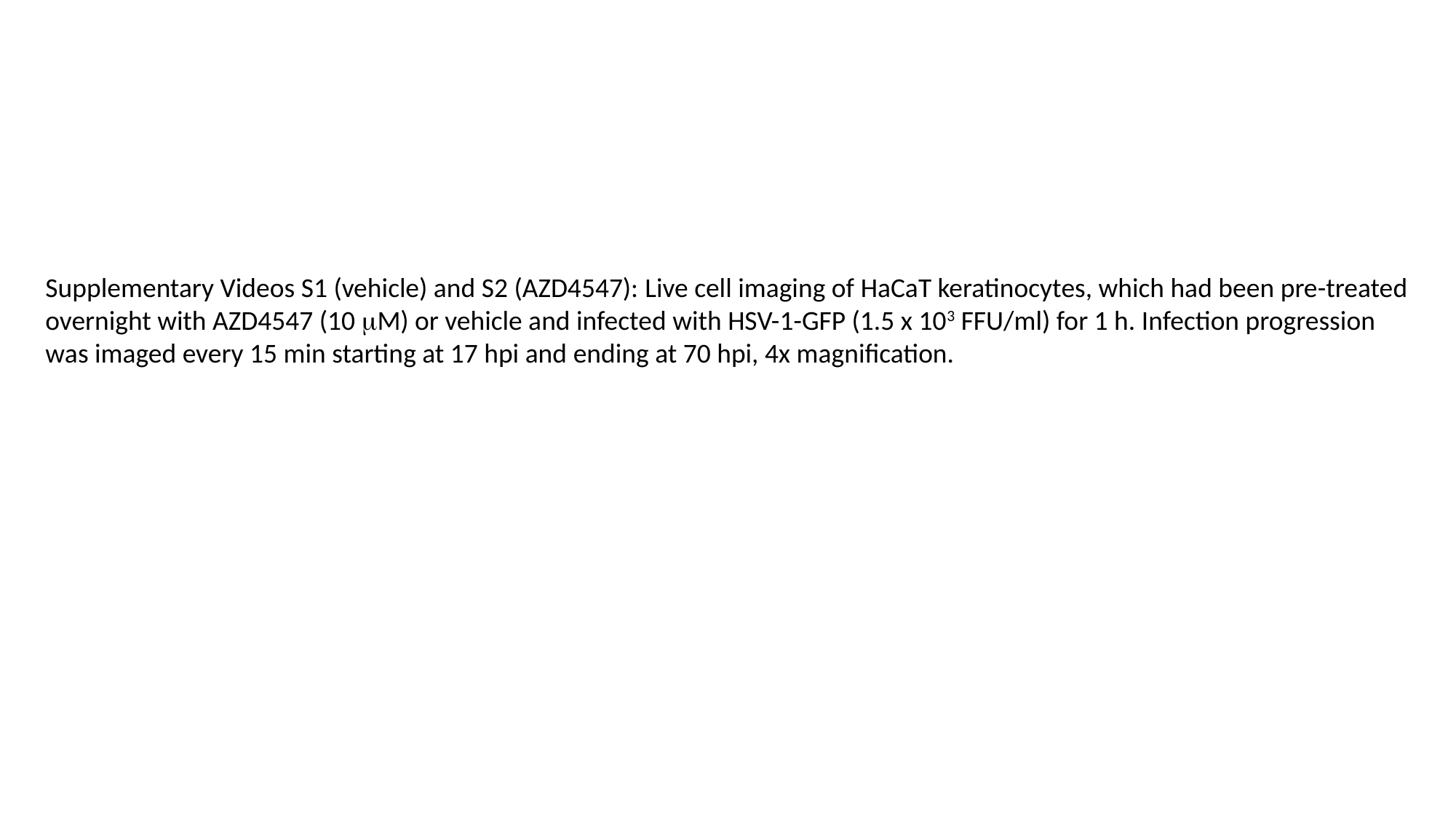

Supplementary Videos S1 (vehicle) and S2 (AZD4547): Live cell imaging of HaCaT keratinocytes, which had been pre-treated overnight with AZD4547 (10 mM) or vehicle and infected with HSV-1-GFP (1.5 x 10^3^ FFU/ml) for 1 h. Infection progression was imaged every 15 min starting at 17 hpi and ending at 70 hpi, 4x magnification.
