# Supplementary material for: FGF receptor kinase inhibitors exhibit broad antiviral activity by targeting Src family kinases
Source: Cell Mol Life Sci. 2024 Dec 2;81(1):471. doi: 10.1007/s00018-024-05502-x (PMC11612106; doi:10.1007/s00018-024-05502-x)
Supplement: Supplementary file 2 — Supplementary Material 2 [file 18_2024_5502_MOESM2_ESM.docx]

###
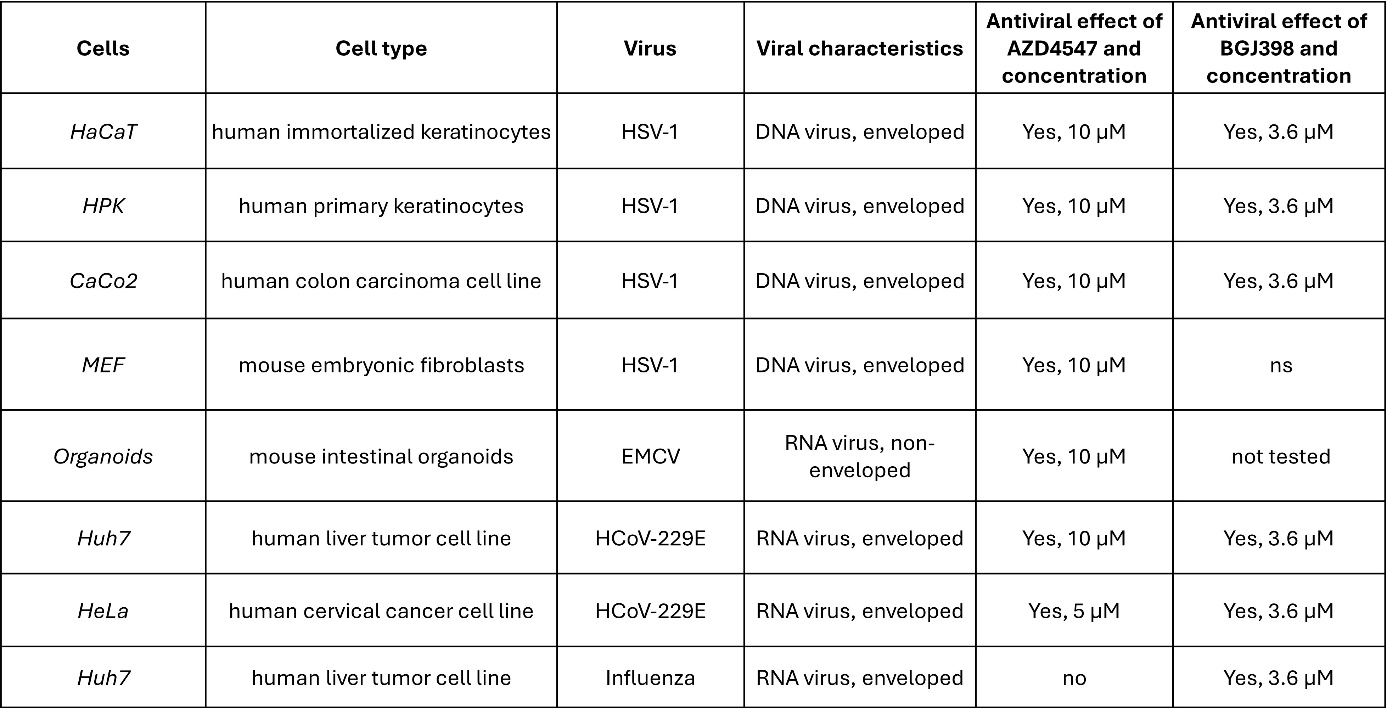


**Table S1: Summary of the antiviral activity of AZD4547 and BGJ398 in the different infection models used in the experiments shown in Fig. 1 and Fig. 2.**


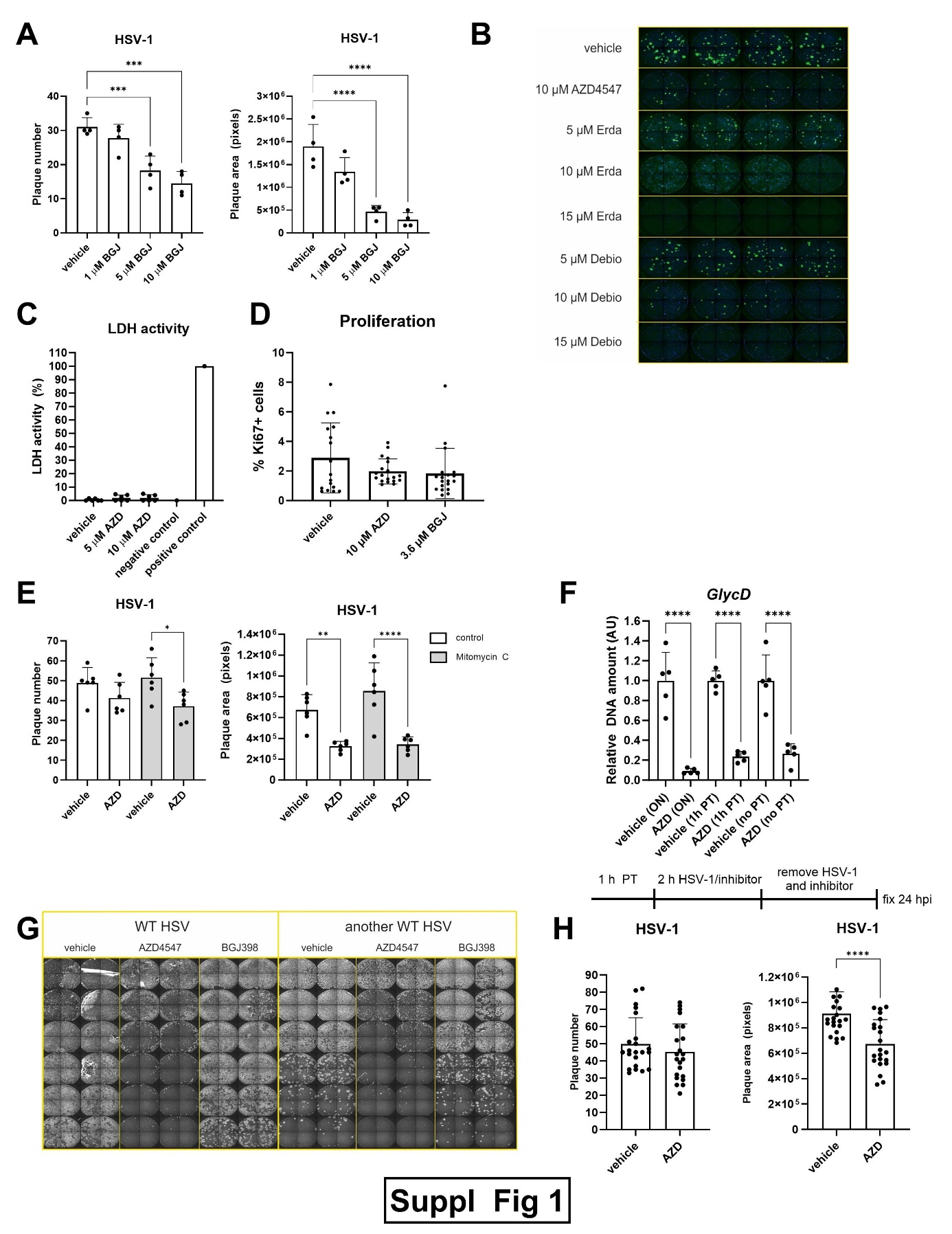


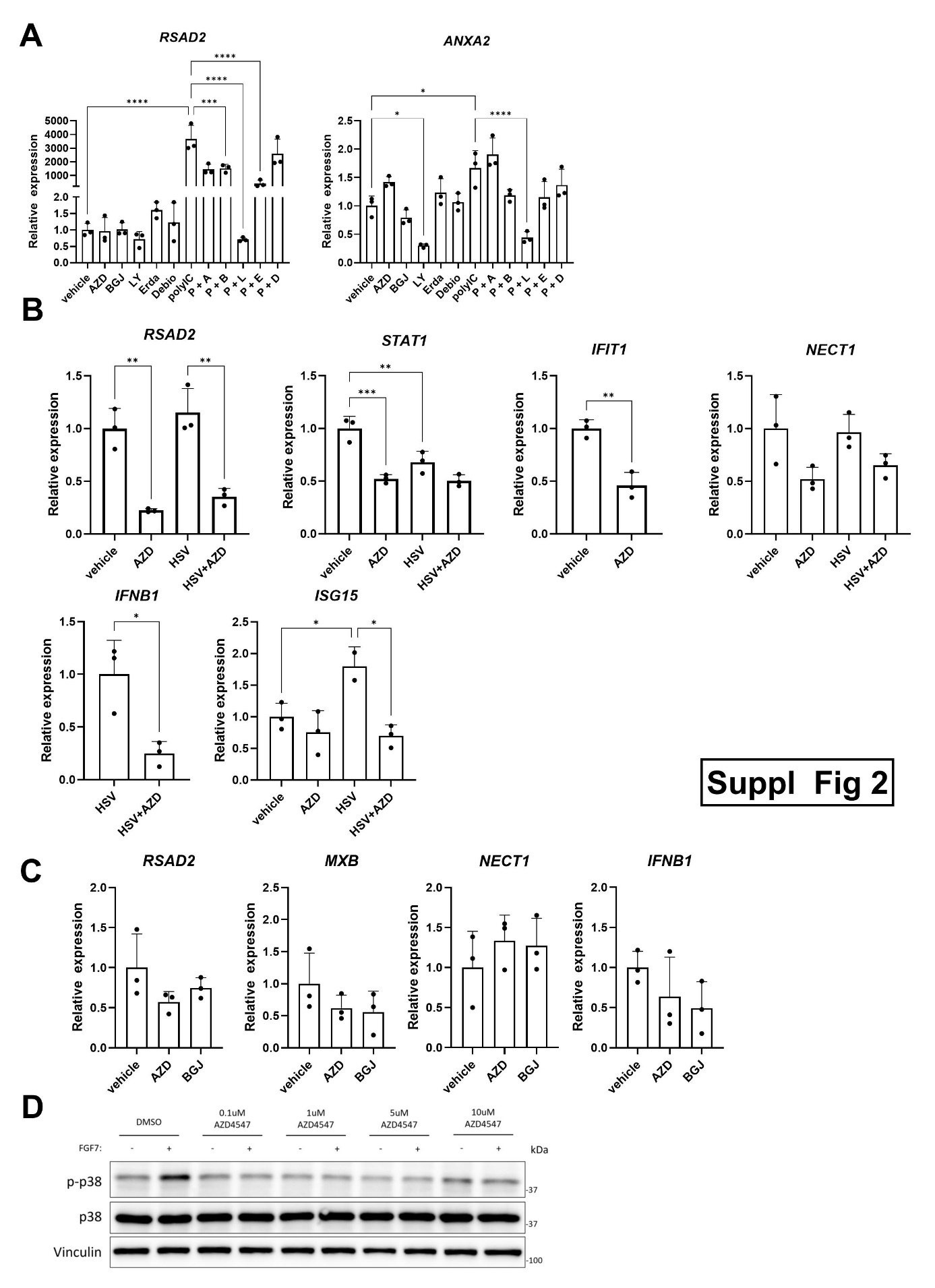


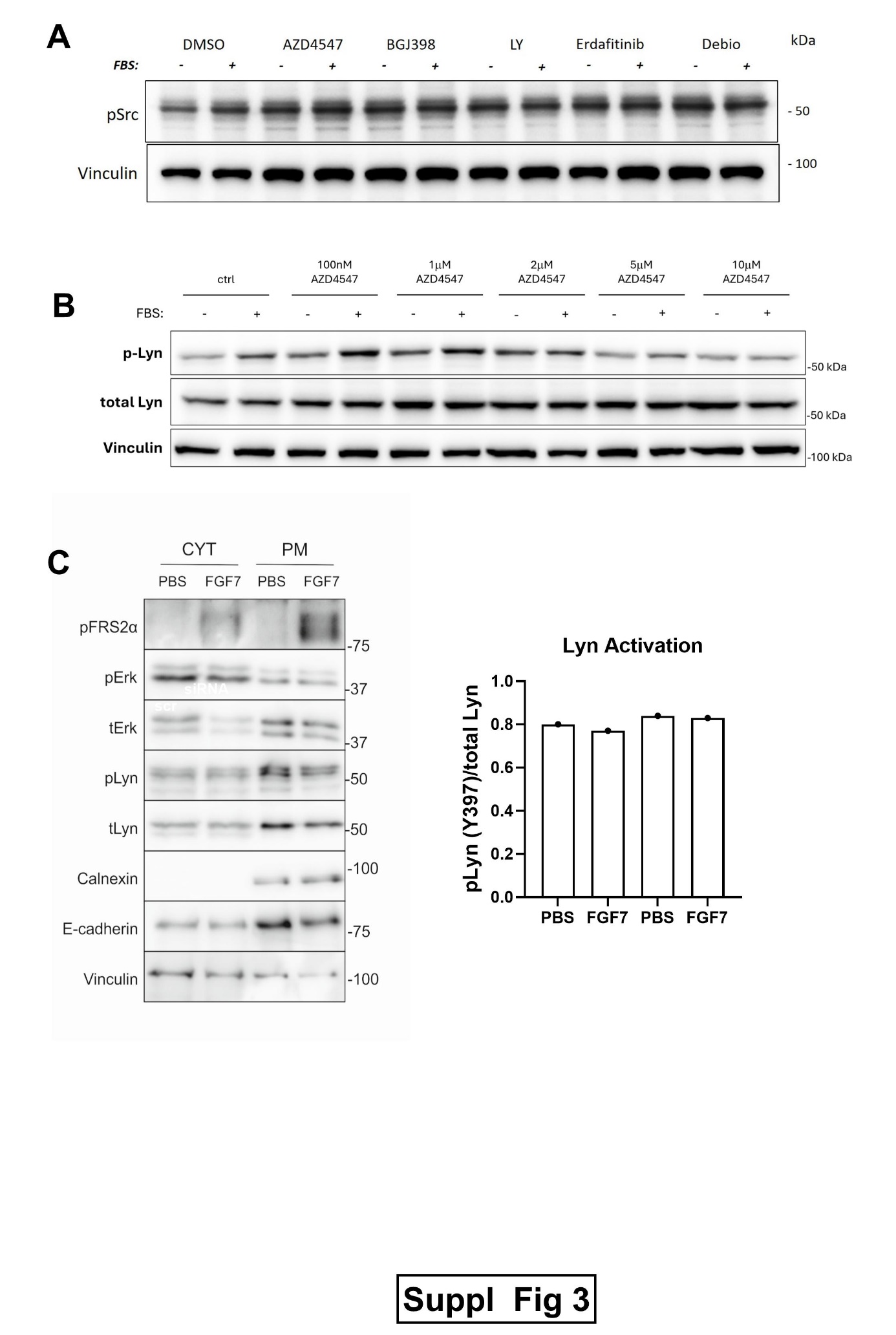

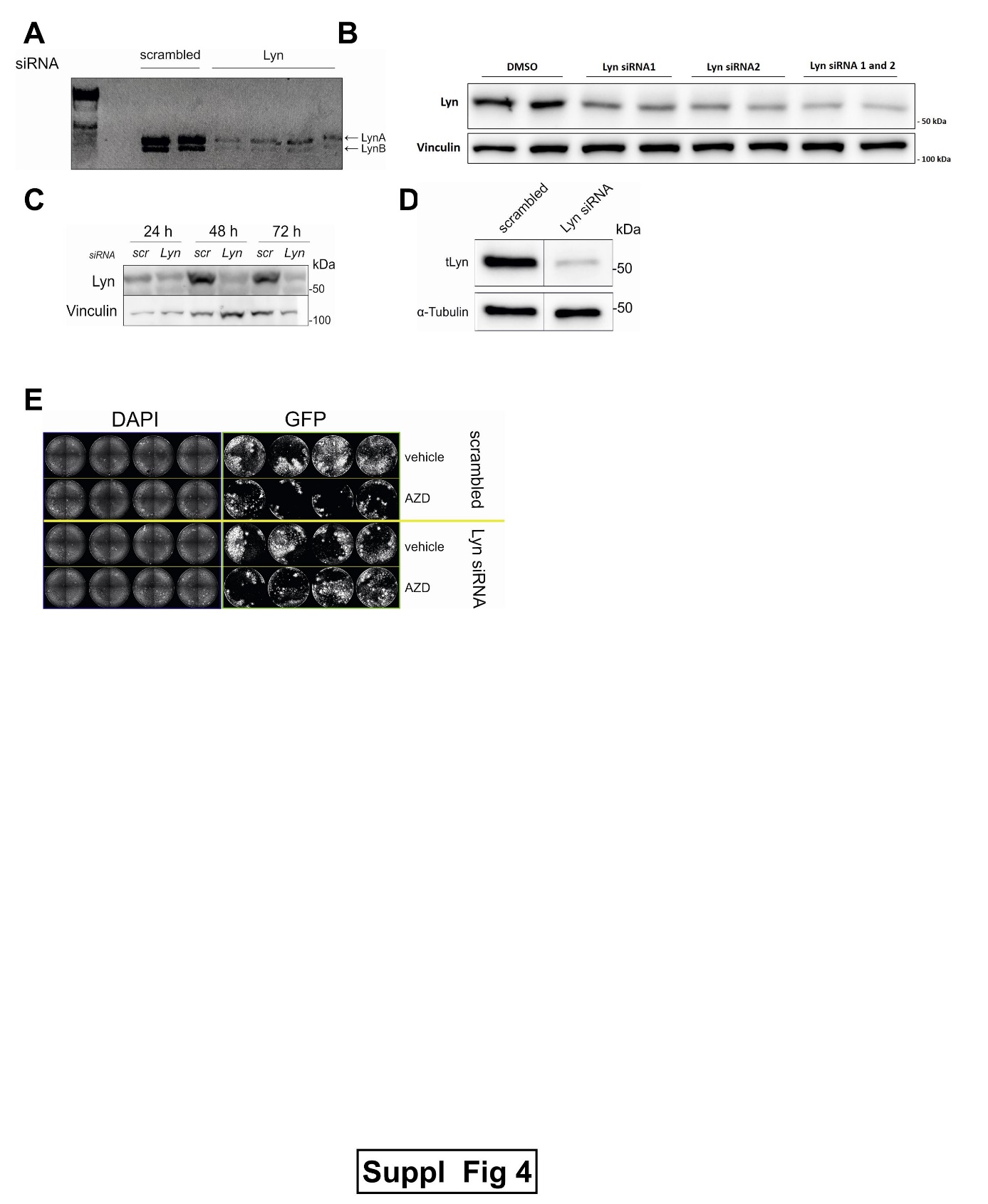


**Supplementary Figure Legends**

**Figure S1: FGFR inhibitors exhibit antiviral activity against HSV-1**

**A:** Plaque number and area at 24 hpi of HaCaT keratinocytes, which had been pre-treated for 1 h with different concentrations of BGJ398 or vehicle and infected for 1 h with HSV1-GFP (1.5 x 10^3^ FFU/ml).

**B:** Fluorescence imaging of HaCaT keratinocytes, which had been pre-treated ON with AZD4547 (10 µM), Erdafitinib (5-15 µM), Debio-1347 (5-15 µM) or vehicle and infected for 1 h with HSV-1-GFP (1.5 x 10^3^ FFU/ml). Fluorescence was analyzed at 24 hpi.

**C:** Lactate dehydrogenase (LDH) activity in the supernatant of HaCaT keratinocytes after treatment with AZD4547 (10 µM) or vehicle for 24 h. LDH activity in the cell lysates was used as positive control.

**D:** Percentage of Ki67-positive HaCaT keratinocytes after treatment with AZD4547 (10 µM), BGJ398 (3.6 µM) or vehicle for 24h; determined by immunofluorescence staining.

**E:** Plaque number and area at 24 hpi of HaCaT keratinocytes, which had been infected with HSV-1-GFP (1.5 x 10^3^ FFU/ml µg/ml) for 1 h and treated with a combination of DMSO or 10 µM AZD4547 and vehicle or Mitomycin C (0.2 µg/ml).

**F:** qPCR for *GlycD* relative to *ACTB* using DNA from HaCaT keratinocytes, which had been pre-treated ON or for 1 h with DMSO or 10 µM AZD4547 and infected with HSV-1 (1.8 x 10^5^ PFU/ml).

**G:** Representative immunofluorescence stainings of HaCaT keratinocytes, which had been pre-treated ON with AZD4547 (10 µM), BGJ398 (3.6 µM) or vehicle and infected for 1 h with serial dilutions of two different strains of WT-HSV-1. Staining with an antibody against purified DNA-containing HSV-1 capsids was performed at 24 hpi.

**H:** Plaque number and area at 24 hpi of HaCaT keratinocytes, which had been pre-treated for 1 h with AZD4547 (10 µM) or vehicle and infected with HSV-1-GFP (1.5 x 10^3^ FFU/ml) for 2 h in the presence of the inhibitor or DMSO. After removal of the virus and two washing steps, cells were incubated in DMEM/5% FBS without DMSO or AZD4547.

Bar graphs show mean +/- SD. *P<0.05, **P<0.01, ***P<0.001, ****P<0.0001. A, D-F: One-way ANOVA, H: Unpaired t-test. N (biological replicates) = 4 from 1 experiment (A), N=6 from 2 experiments (C), N=17-19 from 2 experiments (D), N=6 from 2 experiments (E) and N=5 from 2 experiments (F), N=22 from 4 experiments (H).

**Figure S2: The antiviral effect of FGFR inhibitors is not mediated through regulation of ISG expression**

**A:** qRT-PCR for *RSAD2* and *ANXA2* relative to *RPL27* using RNA from HaCaT keratinocytes, which had been serum-starved ON, pre-treated for 1 h with AZD4547 (10 µM), BGJ398 (3.6 µM), Erdafitinib or Debio-1347 (5 µM each) or vehicle and then incubated with poly(I:C) for 6 h in DMEM/5% FBS.

**B:** qRT-PCR for different ISGs and *IFNB1* relative to *RPL27* using RNA from HaCaT keratinocytes, which had been pre-treated ON with AZD4547 (10 µM) or vehicle and then infected for 7 h with HSV1-GFP.

**C:** qRT-PCR for different ISGs and *IFNB1* relative to *RPL27* using RNA from HaCaT keratinocytes, which had been infected with HSV1-GFP (7.3 x 10^4^ FFU/ml), treated with AZD4547 (10 µM), BGJ398 (3.6 µM) or vehicle at 7 hpi and lysed after ON incubation.

**D:** Western blot analysis for phosphorylated and total p38 and for vinculin using lysates from HaCaT keratinocytes, which had been serum-starved overnight, treated for 1 h with different concentrations of AZD4547 and incubated for 10 min with 10 ng/ml FGF7 or vehicle.

Bar graphs show mean +/- SD. *P<0.05, **P<0.01, ***P<0.001, ****P<0.0001. A-C: One-way ANOVA. N (biological replicates) = 3 from 1 experiment (A-C).

**Figure S3: FGFR inhibitors do not affect Src activity, and FGF7 does not affect the subcellular location of Lyn**

**A:** Western blot analysis for pSrc-Tyr416 and vinculin using lysates from HaCaT keratinocytes, which had been serum-starved ON, pre-treated for 1 h with AZD4547 (10 µM), BGJ398 (3.6 µM), Erdafitinib or Debio-1347 (5 µM each) and incubated for 15 min with 5% FBS.

**B:** Western blot analysis for pLyn-Tyr397, total Lyn and vinculin in HaCaT keratinocytes, which had been starved ON, pre-treated for 1 h with different concentrations of AZD4547 or with vehicle (DMSO) and stimulated for 15 min with 10% FBS.

**C:** Western blot analysis for pFRS2α, phosphorylated and total ERK and Lyn, calnexin, E-cadherin and vinculin using cytoplasmic (Cyt) and plasma membrane (PM) lysates from HaCaT keratinocytes, which had been starved ON and incubated with FGF7 (10 ng/ml) for 15 min. Quantification of the band intensities is shown in the graph.

**Figure 4: Lyn knock-down efficiency and role of Lyn in the antiviral activity of AZD4547**

**A:** Agarose gel electrophoresis of the Lyn RT-PCR products of RNA of HaCaT cells, which had been transfected with scrambled siRNA (first two lanes) or Lyn siRNA (last four lanes). Bands corresponding to Lyn A and Lyn B isoforms are indicated.

**B:** Western blot analysis for Lyn and vinculin using lysates from HaCaT keratinocytes cultured in a 6-well plate, which had been transfected with scrambled siRNA or two different Lyn siRNAs and lysed at 48 h after transfection.

**C:** Western blot analysis for Lyn and vinculin using lysates from HaCaT keratinocytes, which had been transfected with scrambled (scr) siRNA or a mix of two different Lyn siRNAs and lysed at 24, 48 or 72 h after transfection.

**D:** Western blot analysis for Lyn and α-tubulin using lysates from HaCaT keratinocytes, which had been transfected with scrambled (scr) siRNA or a mix of two different Lyn siRNAs and lysed at 48 h after transfection.

**E:** Representative image at 48 hpi from HaCaT keratinocytes, which had been transfected with scrambled or Lyn siRNA, infected at 48-60 h post transfection with HSV-1-GFP (1.5 x 10^3^ FFU/ml) for 1 h and incubated in medium with AZD4547 (10 µM) or vehicle. Cells were cultured in a black 96-well plate for imaging.
